# Cholinergic regulation of dendritic Ca^2+^ spikes controls firing mode of hippocampal CA3 pyramidal neurons

**DOI:** 10.1073/pnas.2321501121

**Published:** 2024-11-06

**Authors:** Noémi Kis, Balázs Lükő, Judit Herédi, Ádám Magó, Bela Erlinghagen, Mahboubeh Ahmadi, Snezana Raus Balind, Mátyás Irás, Balázs B. Ujfalussy, Judit K. Makara

**Affiliations:** ^a^Laboratory of Neuronal Signaling, Hungarian Research Network Institute of Experimental Medicine, Budapest 1083, Hungary; ^b^Doctoral College of Semmelweis University, János Szentágothai Neurosciences Division, Budapest 1085, Hungary; ^c^Laboratory of Cellular Neurophysiology, Hungarian Research Network Institute of Experimental Medicine, Budapest 1083, Hungary; ^d^Laboratory of Biological Computation, Hungarian Research Network Institute of Experimental Medicine, Budapest 1083, Hungary

**Keywords:** hippocampus, CA3 pyramidal neurons, dendritic Ca^2+^ spike, cholinergic regulation

## Abstract

Active dendritic integrative mechanisms such as regenerative dendritic spikes enrich the information processing abilities of neurons and fundamentally contribute to behaviorally relevant computations. Dendritic Ca^2+^ spikes are generally thought to produce plateau-like dendritic depolarization and somatic complex spike burst (CSB) firing, which can initiate rapid changes in spatial coding properties of hippocampal pyramidal cells (PCs). However, here we reveal that a morpho-topographically distinguishable subpopulation of rat and mouse hippocampal CA3PCs exhibits compound apical dendritic Ca^2+^ spikes with unusually short duration that do not support the firing of sustained CSBs. These Ca^2+^ spikes are mediated by L-type Ca^2+^ channels and their time course is restricted by A- and M-type K^+^ channels. Cholinergic activation powerfully converts short Ca^2+^ spikes to long-duration forms, and facilitates and prolongs CSB firing. We propose that cholinergic neuromodulation controls the ability of a CA3PC subtype to generate sustained plateau potentials, providing a state-dependent dendritic mechanism for memory encoding and retrieval.

## Significance

Converging evidence suggests that pyramidal cells in the hippocampal CA3 region (CA3PCs) play a crucial role in memory storage and recall, but the state-dependent cellular mechanisms required to accomplish these tasks are unknown. In CA1PCs, long-duration dendritic Ca^2+^ spikes and the associated bursts can drive rapid synaptic plasticity and change neuronal tuning properties, providing a basis for memory storage. However, Ca^2+^ spikes in CA3PCs are less well explored. Here, we report that rodent CA3PCs show large heterogeneity in dendritic Ca^2+^ spike kinetics under baseline conditions, with a subpopulation of cells firing only unique short-duration Ca^2+^ spikes. However, these short Ca^2+^ spikes are substantially lengthened by acetylcholine (a neuromodulator orchestrating memory processes), suggesting a state-dependent dendritic mechanism for information storage.

Active dendritic processing of synaptic information plays a critical role in neuronal computations. Cortical pyramidal cells (PCs) exhibit various regenerative dendritic spike forms mediated by voltage-gated Na^+^ (VGNC) or Ca^2+^ (VGCC) channels or NMDA receptors (NMDARs), which allow amplification of specific synaptic input patterns and shape the somatic action potential (AP) output. Notably, different types of dendritic spikes have heterogeneous kinetics as measured in vitro, depending on the ion channels mediating the regenerative event: dendritic Na^+^ spikes are very short [~2 to 3 ms (1)], NMDA spikes last long [~30 to >100 ms (2, 3)], whereas Ca^2+^ spikes have a typical duration of ~20 to 60 ms (4, 5). Ca^2+^ spikes (complemented by NMDAR activation) are thought to provide a major component underlying dendritic plateau potentials that can evoke a characteristic firing pattern, complex spike bursts (CSBs) at the soma (6–11). Plateau potentials have recently emerged to the forefront of attention, as studies demonstrated that these large and sustained depolarizing events can induce rapid synaptic plasticity (termed behavioral time scale plasticity, BTSP), and in spatially navigating mice they can elicit instantaneous formation of new place fields in PCs of the hippocampal CA1 area (CA1PCs) (12–14). Interestingly, PCs of the CA3 area (CA3PCs) have higher incidence of long bursts than those in CA1 despite overall lower firing rates (15), and recent results indicate that place field-inducing plateau potentials and CSBs in CA3PCs are particularly prolonged, lasting for up to several hundreds of milliseconds (16). However, the dendritic mechanisms enabling such long-lasting regenerative events in CA3PCs are not well elucidated.

Unlike most cortical PC types endowed with one main long and thick primary apical dendrite, CA3PCs typically have an extensively branching apical dendritic tree with thinner higher-order dendrites ascending from one to five short primary apical trunk dendrites. This arbor structure limited direct investigations of dendritic electrophysiological properties (3, 17–21). Using dendritic patch-clamp recordings we have recently demonstrated (21) that individual higher-order apical dendrites of rat CA3PCs can express distinct forms of Ca^2+^ spikes. One group of Ca^2+^ spikes appears as afterdepolarization following AP(s), can produce CSBs at the soma, and often displays slow time course (>~50 ms halfwidth) after the blockade of VGNCs. In contrast, another Ca^2+^ spike type can be initiated without preceding APs, has fast kinetics (halfwidth <~15 ms), and evokes only single somatic APs. This latter fast Ca^2+^ spike form has not been found in CA1PC dendrites (21), but has been shown in dendrites of layer 2/3 PCs in the human neocortex (22). We also found that different types of Ca^2+^ spikes in CA3PCs could occasionally be evoked at the same dendritic recording site [at different stimulus levels but sporadically also interleaved in a single trace (21)], suggesting diversity of dendritic properties not only across cells but also within the dendritic tree. However, one limitation of our previous investigation was that we examined Ca^2+^ spikes by stimulating only a single trunk dendrite, a minor part of the apical dendritic tree (21). This left several questions open: Are heterogeneous Ca^2+^ spike forms expressed within the dendritic arbor of individual neurons? How do Ca^2+^ spikes of all dendritic compartments combine upon widespread dendritic depolarization to create compound Ca^2+^ spikes and shape the final AP output at the soma? Are all CA3PCs able to generate slow dendritic Ca^2+^ spikes, or is there a subpopulation of neurons lacking the ability to fire slow Ca^2+^ plateaus that could form the basis of prolonged CSBs inducing BTSP?

Integrative functions of dendrites may be drastically influenced by neuromodulation. The ascending cholinergic projection from the basal forebrain modulates cellular and network activity in the hippocampus, and these effects altogether are thought to facilitate the encoding of new information (23, 24). Acetylcholine affects various ion channels and has been shown to enhance dendritic plateau potentials in cortical PCs (25, 26).

Given the primary role attributed to dendritic plateau potentials in the formation of hippocampal spatial representations, here we set out to comprehensively investigate the properties of compound dendritic Ca^2+^ spikes and their regulation by cholinergic activity in CA3PCs.

## Results

### Cell-to-cell Heterogeneity of Compound Dendritic Ca^2+^ Spikes.

**Fig. 1.**
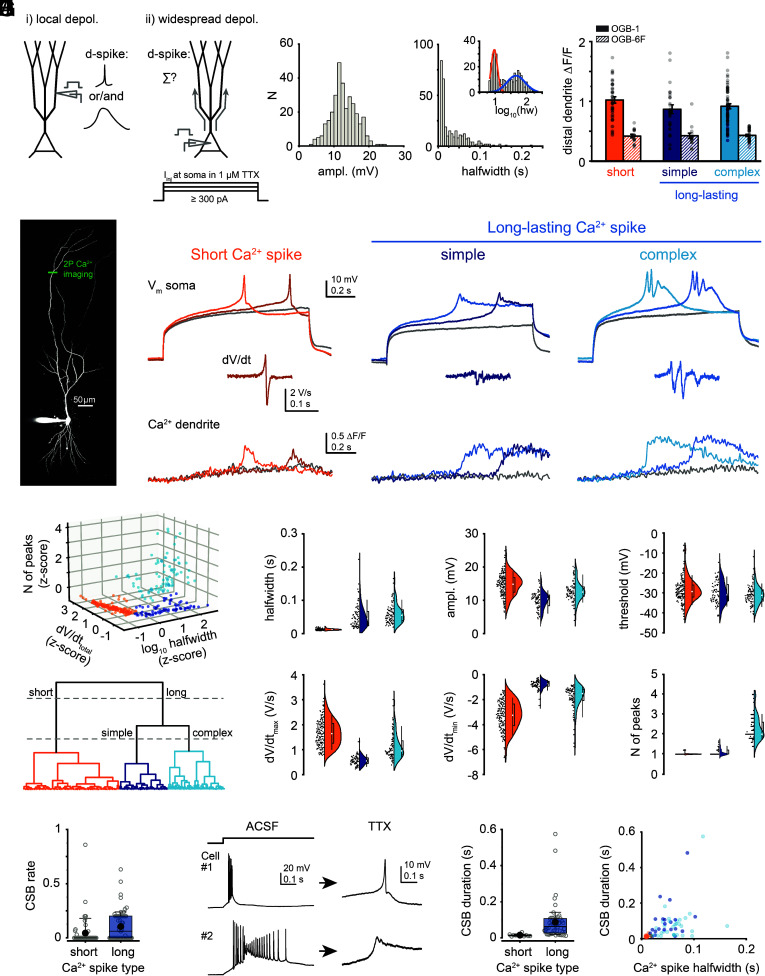
Heterogeneity of compound dendritic Ca^2+^ spikes in CA3PCs (*A*) Schematic illustration of the two types of dendritic Ca^2+^ spikes activated in individual branches (*Left*) and the experimental approach to assess their combined activation (*Right*). (*B*) 2P collapsed z-stack of a CA3PC loaded with 50 μM Alexa Fluor 594 and 100 μM OGB-1. Position of the Ca^2+^ imaging line is indicated in green. (*C*) Experimental protocol to evoke compound Ca^2+^ spikes: 1-s-long somatic current injections (I_inj_) with variable amplitudes were applied in the presence of 1 μM TTX (see also *SI Appendix* Fig. 1). (*D*) Examples of compound Ca^2+^ spike types evoked by somatic I_inj_ in three different CA3PCs. *Top*: somatic voltage response to subthreshold and two different levels of suprathreshold I_inj_; *Middle,* dV/dt of the color-matched Ca^2+^ spike enlarged; *Bottom*, Ca^2+^ signals corresponding to the voltage traces measured on a distal dendrite with OGB-1. (*E*) Distribution of the amplitude (*Left*) and the halfwidth (*Right*) of compound Ca^2+^ spikes (n = 317 cells). The inset shows the log_10_ (halfwidth) values that segregate to two distinct groups. (*F*) Summary of Ca^2+^ spike-associated dendritic Ca^2+^ signal amplitudes measured >300 μm from the soma. Data are shown separately for cells loaded with the high-affinity dye OGB-1 (empty bars) or the low-affinity dye OGB-6F (striped bars). Dots represent individual data points, bar graphs show mean ± SEM (OGB-1 short: n = 59, simple long-lasting: n = 26, complex long-lasting: n = 35, *P* = 0.097; OGB-6F short: n = 33, simple long-lasting: n = 16, complex long-lasting: n = 15, *P* = 0.792, Kruskal–Wallis tests). Distal dendritic Ca^2+^ signals were detected even with OGB-6F. (*G*) Hierarchical cluster analysis. *Top*, individual data points along the three main z-scored parameters; *Bottom*, dendrogram of n = 317 cells (152 short, 76 simple long-lasting, 89 complex long-lasting). (*H*) Distribution of kinetic parameters of Ca^2+^ spikes in all three groups. Statistical analysis showed significant differences between short (n = 152 cells) and long (simple and complex pooled, n = 165 cells) Ca^2+^ spikes (dV/dt_max_: *P* < 0.001; dV/dt_min_: *P* < 0.001; amplitude: *P* < 0.001; halfwidth: *P* < 0.001; threshold: *P* = 0.004; N of peaks: *P* < 0.001; Mann–Whitney test). Note the small effect size for threshold comparison (1.84 mV difference). (*I*) CSB rate (at 600 pA I_inj_) in cells with short (n = 62) and long-lasting (n = 75) Ca^2+^ spikes (*P* = 0.002, Mann–Whitney test). Gray open circles: individual cells; box: interquartile interval; line: median; black filled circle: mean; whiskers: 10 to 90%. (*J*) *Left*, examples of CSBs with different kinetics in ACSF and the corresponding Ca^2+^ spike types after TTX application (segments cut from 1-s-long I_inj_ steps). *Middle*, CSB duration in cells with short (n = 11) and long-lasting (n = 52) Ca^2+^ spikes (*P* < 0.001, Mann–Whitney test). Box plot as in *I*. *Right*, relationship between CSB duration and Ca^2+^ spike halfwidth (n = 63, Spearman R = 0.564, *P* < 0.001).

Based on our previous work (19, 21) we hypothesized that widespread depolarization of the apical dendritic tree — recruiting Ca^2+^ spikes with potentially diverse kinetics in different dendritic domains — can result in compound Ca^2+^ spikes with variable properties that may be dominated by either fast or slow components (Fig. 1*A*).

To study the properties of such compound dendritic Ca^2+^ spikes, we patched CA3PCs at the soma in acute slices from adult rats, and recorded voltage responses and apical dendritic Ca^2+^ signals (Fig. 1*B*) elicited by 1-s-long step (Fig. 1*C*) or ramp (*SI Appendix*, Fig. S1*A*) current injections in the presence of the voltage-gated Na^+^ channel blocker tetrodotoxin (TTX, 1 μM). Under these conditions all-or-none regenerative Ca^2+^ spikes could be evoked in the majority of CA3PCs (19) (Fig. 1*D*, 331 of 361 cells, 91.7 %). The Ca^2+^ spike voltage waveforms were stereotypical within a given CA3PC regardless of the level of I_inj_ (Fig. 1*D* and *SI Appendix,* Fig. S1*B*), but they were highly variable from cell to cell (Fig. 1 *D* and *E* and *SI Appendix*, Fig. S1 *B* and *C*). While spike amplitude was distributed normally in the cell population (Fig. 1*E*, n = 317, *P* = 0.416, Shapiro–Wilks test for normality), kinetic parameters such as the rate of rise (dV/dt_max_), rate of decay (dV/dt_min_), and duration (measured as the halfwidth) showed non-Gaussian distribution and large coefficient of variation (CV; Fig. 1*E* and *SI Appendix,* Fig. S1*C*). The most heterogeneous parameter was Ca^2+^ spike halfwidth (CV = 0.984; *P* < 0.001, Shapiro–Wilks test for normality) that clearly separated the cells into two main groups (Fig. 1*E*, *Right*): one group of CA3PCs had Ca^2+^ spikes with a very short halfwidth (peak of halfwidth at 9.44 ms), whereas another group of CA3PCs expressed long-lasting Ca^2+^ spikes (peak of halfwidth at 46.24 ms). In addition, long-lasting Ca^2+^ spikes also displayed considerable heterogeneity, and in many cells appeared as a complex, multipeak Ca^2+^ spike phenotype, representing a mixture of slow and superimposed fast components that were repetitively activated at 10 to 70 Hz frequency (Fig. 1*D* and *SI Appendix,* Fig. S1*B*, 35.3 ± 1.3 Hz, n = 89).

While the Ca^2+^ spikes were evoked by I_inj_ at the soma, both short and long-duration events recruited regenerative Ca^2+^ channel activation throughout the apical dendritic arbor. This was supported by two lines of evidence. First, the spikes were accompanied by time-locked Ca^2+^ signals of similar amplitudes in distal apical dendrites (>300 μm from soma) in all Ca^2+^ spike groups (Fig. 1 *D* and *F*), and Ca^2+^ signals co-occurred in simultaneously recorded dendrites that belonged to different dendritic subtrees (*SI Appendix,* Fig. S1*D*). Second, in dual recordings from soma and a higher-order apical dendritic trunk (distance from soma: 274 ± 23 μm, n = 8) both short and long Ca^2+^ spikes evoked by somatic I_inj_ had larger amplitude and rate of rise in the dendrite than at the soma (dendrite/soma ratio, amplitude: 1.66 ± 0.16, *P* = 0.011; dV/dt_max_: 1.63 ± 0.16, *P* = 0.011, one-sample Wilcoxon test), while the kinetic profile was similar (*SI Appendix,* Fig. S2 *A*–*C*). Although the site of Ca^2+^ spike initiation could not be precisely determined from the above experiments (*SI Appendix,* Fig. S2*D* and *SI Appendix*, *Supplemental Discussion*), the results are consistent with widespread engagement of regenerative Ca^2+^ responses in the apical dendrites.

Hierarchical clustering using the most distinctive Ca^2+^ spike features (dV/dt_total_, spike duration, and number of peaks; *SI Appendix.* Fig. S1*E*) confirmed that the observed heterogeneity in Ca^2+^ spike waveforms could be well described by three clusters: a first division of the dendrogram produced short- and long-lasting Ca^2+^ spike groups, and the latter group was further subdivided into simple and complex forms (Fig. 1*G*, n = 317 cells). Short Ca^2+^ spikes had on average steeper rise and decay than long-lasting Ca^2+^ spikes, whereas their amplitude and threshold were relatively similar (Fig. 1*H*). The segregation to three clusters suggests that distinct compound Ca^2+^ spike patterns are produced by different contributions of fast and slow Ca^2+^ spikes in individual apical trunk dendrites of CA3PCs, with a subpopulation of CA3PCs where dendrites apparently express dominantly fast Ca^2+^ spikes.

Ca^2+^ spikes have been shown to underlie CSB firing at the soma (7, 10, 19). To address how the different Ca^2+^ spike types are related to CSB properties, in a subset of cells we first measured CSB rate and duration under control conditions in ACSF, and then washed in TTX to eliminate APs allowing determination of the Ca^2+^ spike type based on the above kinetic parameters. CA3PCs expressing short compound Ca^2+^ spikes had low or zero propensity to fire CSBs and were mostly regular spiking [RS cells (19); *SI Appendix*], and when they did fire CSBs (typically at high I_inj_), the bursts had short duration (Fig. 1 *I* and *J*). In contrast, cells exhibiting long-lasting Ca^2+^ spikes had higher CSB propensity (mostly CSB cells) and produced longer, plateau-like CSBs (Fig. 1 *I* and *J*). Furthermore, while Ca^2+^ spike duration was significantly shorter in RS than in CSB cells, the amplitude and dV/dt_max_ of Ca^2+^ spikes were similar between the two firing pattern categories, further emphasizing the importance of Ca^2+^ spike kinetics rather than magnitude (*SI Appendix,* Fig. S3).

Altogether the above results suggest that CA3PCs are composed of functional subtypes with distinct firing modes depending on the dominance of fast or slow Ca^2+^ spikes. Remarkably, under baseline conditions in the slice, a sizable fraction of CA3PCs expresses extremely fast compound Ca^2+^ spikes that per se do not sustain prolonged plateau potentials.

### Morpho-Topographic Mapping of Ca^2+^ Spike Heterogeneity.

**Fig. 2.**
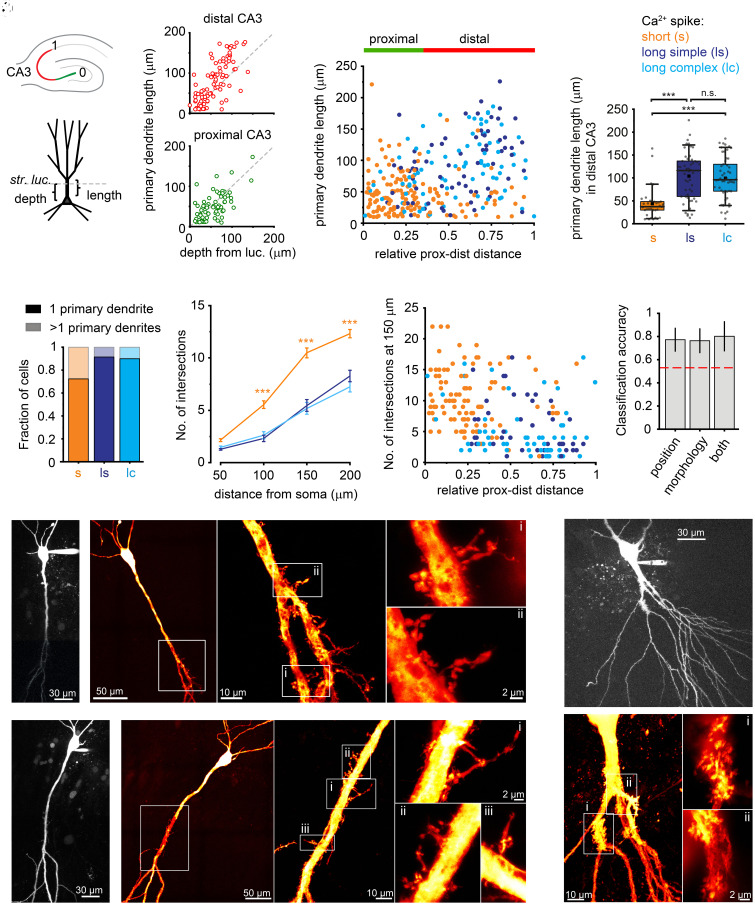
Morpho-topographic correlates of Ca^2+^ spike heterogeneity (*A*) Schematic showing the measurement of proximodistal position (*Top*) and primary apical trunk length (*Bottom*). (*B*) Correlation of average primary apical trunk length and soma depth from the border of str. lucidum in distal (red, relative proximodistal position: 0.4 to 1; Spearman R = 0.748, *P* < 0.001, n = 94) and proximal (green, relative proximodistal position ≤0.3; Spearman R = 0.556, *P* < 0.001, n = 68) CA3PCs. Note that the deepest distal CA3PCs often have particularly long primary trunks due to late bifurcation in str. lucidum. (*C*) Distribution of the Ca^2+^ spike clusters with relative proximodistal position along CA3 and with mean primary apical dendrite length. Dots represent individual cells (n = 298) color-coded for clusters (orange: short; deep blue: simple long-lasting; light blue: complex long-lasting). (*D*) Difference in primary apical dendrite length between CA3PCs with short and long-lasting Ca^2+^ spikes in the distal CA3 subregion. Kruskal–Wallis test: *P* < 0.001; post hoc multiple comparisons test: short vs simple long: *P* < 0.001, short vs complex long: *P* < 0.001, simple long vs complex long: *P* = 1. (*E*) Fraction of cells with 1 or more than 1 primary dendrites. Data are shown separately for cells with different Ca^2+^ spike types. (short (s): n = 148, long simple (ls): n = 70, long complex (lc): n = 80). (*F*) Number of apical dendritic intersections at different distances from the soma (Sholl analysis, n = 211 cells). Mixed ANOVA: *P* < 0.001 for distance, *P* < 0.001 for cluster, *P* < 0.001 for interaction. Post hoc Tukey’s test indicates difference between short vs long simple and short vs long complex cells (***P < 0.001) at 100, 150, and 200 μm distances, but no significant difference at any distance between the two long clusters (*P* > 0.890 for all comparisons). (*G*) Number of apical dendritic intersections at 150 μm from the soma as a function of the relative proximodistal position along CA3. Colors indicate the Ca^2+^ spike cluster label of the neurons (n = 211 cells, color code as in C). (*H*) Classification accuracy (mean and SD across 10-fold cross validation groups; *SI Appendix*) for predicting Ca^2+^ spike type (short- or long-duration) based on topographic position (proximodistal position along CA3 and radial depth from str. lucidum), neuronal morphology (number and length of trunk, Sholl intersections), or both (n = 132 cells). There is no significant difference between the groups (Friedman test, *P* = 0.52). The red dashed line represents chance level. (*I*) 2P z-stack of a long-shafted deep distal CA3PCs filled with Alexa Fluor 594 and biocytin. (*J*) Confocal maximum intensity projection images of the cell in I. White box on the *Left* indicates the location of the enlarged apical dendritic area on the *Right*. Note the thorny excrescences along the trunk. Boxed areas are shown at higher magnification in *K*. (*K*) STED microscopy images of thorny excrescences of the cell in *I* and *J*. (*L*–*N*) Same as *I*–*K* for another long-shafted deep distal CA3PCs filled with Alexa Fluor 594 and biocytin. (*O*) 2P z-stack of a short-shafted proximal CA3PCs filled with Alexa Fluor 594 and biocytin. (*P*) Confocal image of the apical trunk of the cell in *O*. Note the clusters of TEs on the primary and secondary apical trunks. Boxed areas are shown at higher magnification in *Q*. (*Q*) STED microscopy image sections of thorny excrescence clusters of the cell in *O* and *P*.

Are there any particular anatomical or morphological characteristics of CA3PCs with different Ca^2+^ spike forms? CA3PCs exhibit considerable heterogeneity of electrophysiological, morphological, genetic, and network properties along the proximodistal [from dentate gyrus (DG) to CA2] and the radial (from deep to superficial pyramidal cell layers) axes of CA3 (19, 27–34) (Fig. 2*A*). In particular, the radial soma depth is generally proportional to the length of the primary apical dendrites throughout the CA3 area (19, 29, 30) (Fig. 2*B*), suggesting that it impacts dendritic electrophysiology. We therefore determined how the different Ca^2+^ spike clusters mapped onto the proximodistal position of the cell and the length of the primary apical dendrite(s). This mapping revealed nonuniform distribution of CA3PC subtypes (Fig. 2*C* and *SI Appendix,* Fig. S5*A* for individual spike parameters). Cells with short Ca^2+^ spikes were prominent in proximal CA3, where almost all PCs belonged to this cluster. However, distal from an apparent border at ~0.3 relative position along the full proximodistal extent of CA3 (0-1), long-lasting Ca^2+^ spikes were abundant and the Ca^2+^ spike type was related to dendritic morphology: short Ca^2+^ spikes were mostly restricted to distal PCs with short primary trunks (Fig. 2 *C* and *D*), often heavily decorated with thorny excrescences (TEs) (*SI Appendix,* Fig. S4*A*), whereas distal cells located deeper, especially those with longer apical trunk, expressed long-lasting Ca^2+^ spikes (Fig. 2 *C* and *D*; *SI Appendix,* Fig. S5*B*). These results are in line with the previously described distribution of RS and CSB cells (19) as well as with our previous finding that individual apical dendrites expressing fast dendritic Ca^2+^ spikes were more likely to be observed in cells with short trunk(s) (21).

Next, we analyzed whether different Ca^2+^ spike types are also associated with distinct apical dendritic arborization patterns in the CA3 area. First, we observed that cells with short duration Ca^2+^ spikes often had multiple primary apical trunks (Fig. 2*E* and *SI Appendix,* Fig. S4). Sholl analysis performed at four different distances from the soma (50, 100, 150, 200 μm) revealed that cells with short Ca^2+^ spikes typically displayed a more complex apical dendritic arborization than cells with long-duration Ca^2+^ spikes, as indicated by a higher number of intersections (Fig. 2 *F* and *G*). Furthermore, we found that the Ca^2+^ spike type could be equally well predicted by logistic regression both from topographical position of the somata and from neuronal morphology, further indicating a strong correlation between these variables (Fig. 2*H* and *SI Appendix,* Fig. S5*C*).

A previous report (33) proposed that CA3PCs in young mice and rats are composed of two morpho-functionally distinct subpopulations. One group, termed ‘thorny’ cells, was endowed with TEs indicating innervation by mossy fibers (MFs) and expressed low-frequency firing at rheobase. Another smaller group, termed ‘athorny’ cells, produced bursts at rheobase, were located in deep distal CA3, had long primary dendrites and apparently lacked TEs and MF input. Since this latter subpopulation appears to share properties with cells in deep distal CA3 in our dataset (long primary dendrite, bursting), we carefully examined the presence of TEs in this cell population. Two-photon (2P) imaging revealed at least a few TE-like structures on visualized dendrites in str. lucidum in virtually all of our recorded CA3PCs (see examples in *SI Appendix,* Fig. S4), as reported before (19, 21). Furthermore, stimulated emission depletion (STED) superresolution imaging of biocytin-loaded deep distal CA3PCs with long primary trunk demonstrated characteristic lobular TEs on dendrites near the trunk bifurcation zone in str. lucidum (n = 10 cells, Fig. 2 *I*–*N*), although apparently fewer than that observed on cells in proximal CA3 (Fig. 2 *O*–*Q*, and *SI Appendix,* Fig. S4*C*). While we cannot exclude that some CA3PCs lack TEs entirely, these results suggest that in the adult rat CA3 all subtypes of PCs receive synaptic inputs from MFs, albeit at various densities (see also the Discussion section).

### Ion Channels Underlying Diverse Ca^2+^ Spike Forms.

What are the biophysical factors behind the kinetic variety of Ca^2+^ spikes? Although passive dendritic properties, arising from different dendritic morphologies may play a role, we hypothesized that the variability is more likely produced by differences in ion channels generating or modulating the Ca^2+^ spike.

The first possibility we considered was that distinct types of VGCCs, with different kinetic properties, may be responsible for generating short and long-lasting Ca^2+^ spikes. CA3PCs have been shown to express slow and transient Ca^2+^ currents (35–39). We thus tested whether blockade of different VGCC types (T, L, R, N, P/Q) selectively reduces the amplitude and/or rate of rise of short and long Ca^2+^ spikes.

**Fig. 3.**
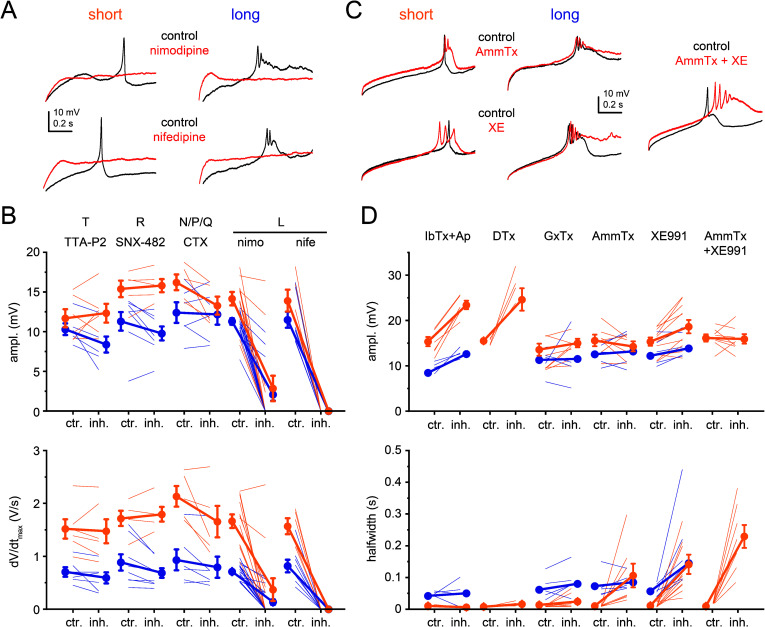
Ion channels underlying diverse Ca^2+^ spike forms (*A*) Example recordings of short (*Left*) and long-lasting (*Right*) Ca^2+^ spikes in TTX before (black control) and 10 to 20 min after (red) bath application of the L-type VGCC inhibitor nimodipine (20 μM, *Top*) or nifedipine (10 μM, *Bottom*). Segments are cut from 1-s-long I_inj_ steps. (*B*) Summary of the effect of various VGCC-type inhibitors on the amplitude (*Top*) and dV/dt_max_ (*Bottom*) of short (orange) and long-lasting (blue) Ca^2+^ spikes. Thin lines represent individual experiments before (ctr) and after (inh) application of VGCC inhibitors; connected symbols show mean ± SEM. Abolished spikes are represented by values of 0. TTA-P2: n = 6 short, n = 6 long spike; SNX-482: n = 5 short, n = 8 long; ω-CTX MVIIC: n = 5 short, n = 6 long; nimodipine: n = 12 short, n = 19 long; nifedipine: n = 5 short, n = 8 long. (*C*) Example recordings of short (*Left*) and long-lasting (*Middle*) Ca^2+^ spikes in TTX, before (black control) and 10 to 20 min after (red) bath application of the A-type VGKC inhibitor AmmTx3 (2 μM, *Top*) or the M-type VGKC inhibitor XE991 (10 μM, *Bottom*). *Right*, combined inhibition of A- and M-type VGKCs in a cell with short Ca^2+^ spike. (*D*) Summary of the effect of various VGKC-type inhibitors on the amplitude (*Top*) and halfwidth (*Bottom*) of short (orange) and long-lasting (blue) Ca^2+^ spikes. Combined iberiotoxin and apamin: n = 6 short, n = 4 long; DTX: n = 4 short; GxTx: n = 8 short, n = 5 long; AmmTx3: n = 7 short, n = 5 long; XE991: n = 11 short, n = 6 long; combined AmmTx3 and XE991: n = 8 short.

We found that the contribution of T-, R-, and N/P/Q-type VGCCs to short or long-lasting Ca^2+^ spikes was minor. The T-type channel inhibitor TTA-P2 (10 μM) or other T-type channel blockers (*SI Appendix,* Fig. S6*D*), and the R-type channel inhibitor SNX-482 (0.5 μM) slightly reduced long Ca^2+^ spikes, while the N/P/Q-type channel inhibitor ω-CTX MVIIC (1 μM) slightly inhibited short Ca^2+^ spikes, but these effects were not robust (Fig. 3 *A* and *B* and *SI Appendix,* Fig. S6 *C* and *D*; see also *SI Appendix,* Fig. S6 *A* and *B* for temporal stability of parameters). The efficacy of TTA-P2, SNX-482, and ω-CTX MVIIC at the applied concentrations has been verified in independent experiments (*SI Appendix,* Fig. S6 *E*–*G*). In contrast to the above blockers, the L-type channel antagonists nimodipine (20 μM) or nifedipine (10 μM) abolished, or strongly reduced the amplitude and dV/dt_max_ of both short and long-lasting Ca^2+^ spikes (Fig. 3 *A* and *B*). We conclude that L-type VGCCs are the dominant channels mediating all types of Ca^2+^ spikes in CA3PCs, and that the large variability of Ca^2+^ spike kinetics cannot be explained by different underlying VGCC types.

We next examined the possibility that the activation of specific K^+^ conductances may produce rapid repolarization of short Ca^2+^ spikes, prelimiting the evolution of a slow event. If this hypothesis is correct, pharmacological inhibition of the responsible K^+^ channel(s) may preferentially prolong fast Ca^2+^ spikes. We ruled out several potential candidates, as the blockade of Ca^2+^-activated K^+^ channels (using a combination of 0.1 μM iberiotoxin and 0.1 μM apamin), Kv1 (using 0.1 μM DTX), and Kv2 (using 0.1 μM guangxitoxin) subunit-containing voltage-gated K^+^ channels (VGKCs) did not prolong the halfwidth of short Ca^2+^ spikes, despite modulating the amplitude and dV/dt of the spike (Fig. 3*D* and *SI Appendix*, Fig. S6 *H–I*). We could however identify two VGKC types whose activity contributed to regulation of Ca^2+^ spike duration (Fig. 3 *C* and *D* and *SI Appendix,* Fig. S6*I*). First, inhibition of the A-type K^+^ current (mediated by Kv4 channels) using AmmTx3 [1 to 2 μM (40)] increased the halfwidth of short Ca^2+^ spikes. Second, inhibition of the M-type K^+^ current (mediated by Kv7 channels) using XE991 (10 μM) amplified the slow component and prolonged all forms of Ca^2+^ spikes, often inducing repetitive short peaks. The combined blockade of A- and M-type currents was even more effective and shifted short Ca^2+^ spikes toward the complex long-lasting phenotype (Fig. 3 *C* and *D* and *SI Appendix,* Fig. S6*I*), indicating that these two current types play an essential role in determining dendritic Ca^2+^ spike type.

### Cholinergic Regulation of Ca^2+^ Spike Kinetics.

**Fig. 4.**
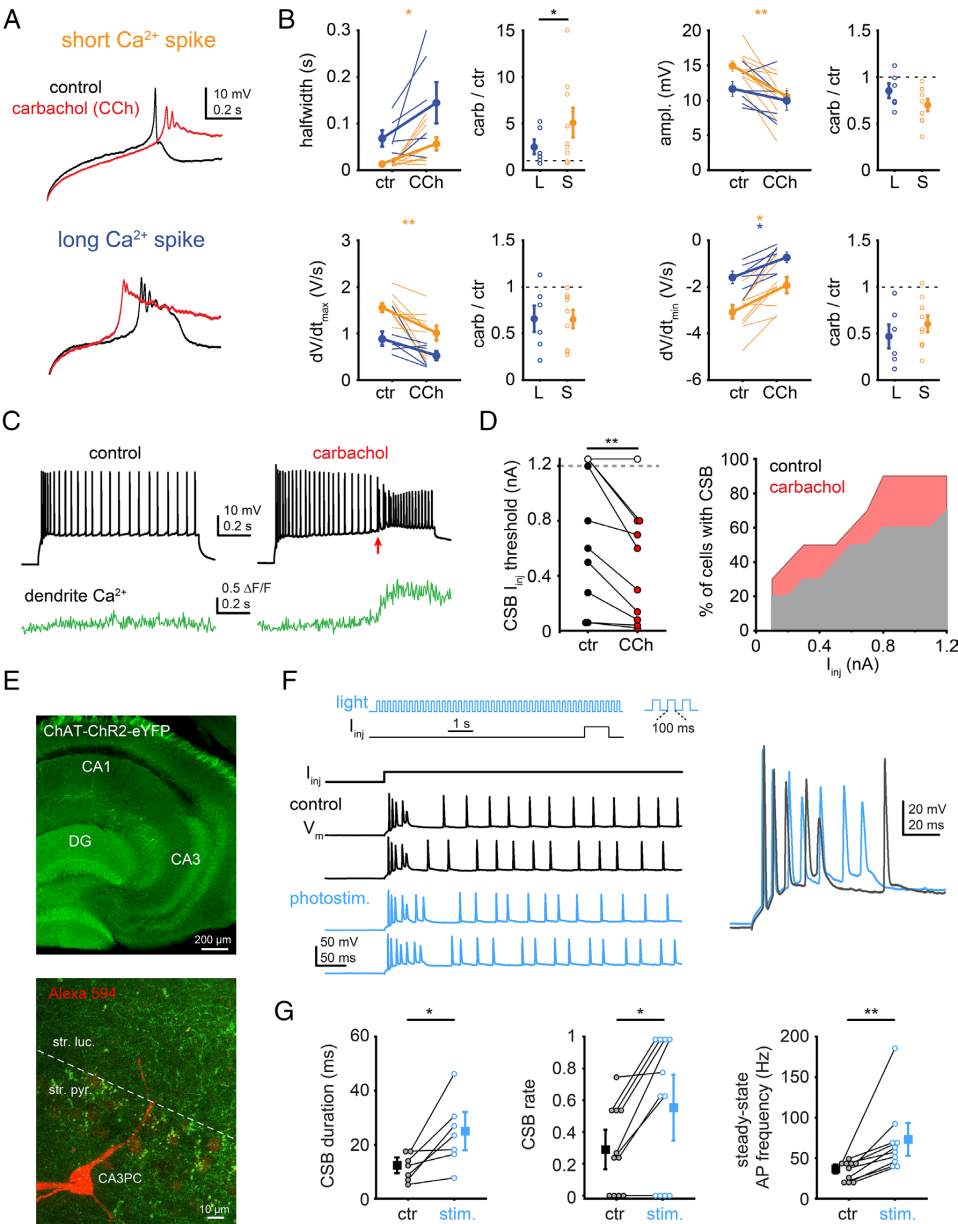
Cholinergic regulation of Ca^2+^ spike kinetics (*A*) Example short (*Top*) and long (*Bottom*) Ca^2+^ spikes in TTX (control, black) and after application of the cholinergic agonist carbachol (CCh; red, 2 μM). (*B*) Effect of carbachol on short (orange, n = 9) and long-lasting (blue, n = 6) Ca^2+^ spike properties. *P < 0.05, **P < 0.01, Wilcoxon test. (*C*) Example recording in ACSF showing firing properties of a CA3PC (*Top*) and distal apical dendritic Ca^2+^ signal (*Bottom*) before (*Left*) and after (*Right*) application of 2 μM carbachol. Note the prolonged CSB (arrow) in the presence of carbachol, also accompanied by distal dendritic Ca^2+^ signal. (*D*) Summary of the effect of carbachol on CSB rate at different levels of I_inj_. *Left*, I_inj_ threshold evoking CSB under control conditions and in carbachol (*P* < 0.01, Wilcoxon test). Symbols connected with lines represent individual experiments. Filled symbols, CSB was evoked at the given I_inj_; open symbols, CSB was not evoked at 1.2 nA I_inj_. *Right*, ratio of CA3PCs (total n = 10 cells) expressing CSB at different I_inj_ levels in control (gray) and in carbachol (red). (*E*) *Top*, widefield fluorescent image of ChR2-eYFP expression in the CA3 area in ChAT-Cre/Ai32 mice. *Bottom*, 2P z-stack of eYFP-ChR2 positive axons (green) surrounding a patched CA3PC loaded with Alexa Fluor 594 (red). (*F*) *Left* and *Top*, protocol of combined photostimulation and I_inj_. *Bottom*, representative voltage responses to somatic I_inj_ in a CA3PC without (black) and with (blue) photostimulation of ChR2. Two traces for CSB comparison are shown overlaid on the *Right*. (*G*) Summary of the effect of photostimulation on CSB duration (*Left*, n = 7 cells with CSB, *P* < 0.05, Wilcoxon test), CSB rate (*Middle*, n = 11, *P* < 0.05, Wilcoxon test), and AP frequency measured at steady-state firing during the 400 to 600 ms segment of the depolarization step (*Right*, n = 10 cells exhibiting regular steady-state AP firing, *P* < 0.01, Wilcoxon test).

Acetylcholine (ACh) released by axons from the medial septum/diagonal band of Broca plays a critical role in learning and memory processes *via* a wide array of intrinsic, synaptic, and network effects (23, 24). Among many other molecular targets (41–43), cholinergic receptor activation modulates the function of voltage-gated ion channels including VGCCs (25, 44–46) and A-type and M-type VGKCs (45, 47) and facilitates bursting (48), raising the idea that CSB firing could be controlled by the cholinergic system via regulating the Ca^2+^ spike profile. We first tested the effect of the nonhydrolyzable cholinergic receptor agonist carbachol on Ca^2+^ spikes in TTX. Strikingly, carbachol (bath-applied at 2 μM concentration) transformed short Ca^2+^ spikes into long-lasting forms, an effect that was reflected not only in ~5-fold increase of the average spike halfwidth but also in reduced amplitude and dV/dt (Fig. 4 *A* and *B*). On the other hand, combined blockade of nicotinic and muscarinic ACh receptors by hexamethonium (100 μM) and ipratropium bromide (10 μM) did not generate an opposite effect, i.e. shortening the long-lasting Ca^2+^ spikes (*SI Appendix,* Fig. S7*A*), indicating that the original heterogeneity of Ca^2+^ spike properties cannot be explained by variable cholinergic tone in the slice. In addition, we tested the effect of carbachol on the firing pattern of CA3PCs in the absence of TTX. Consistent with its effect on Ca^2+^ spikes and in line with previous work (49, 50), carbachol facilitated CSB firing (without signs of network bursts) in response to I_inj_ as well as in response to synaptic stimulation at spines on an apical trunk by 2P glutamate uncaging (2PGU), often inducing sustained bursts and persistent firing (Fig. 4 *C* and *D* and *SI Appendix,* Fig. S7 *B* and *C*). The CSBs triggered in carbachol were eliminated by nifedipine (10 μM), confirming that they were mediated by L-type channels (*SI Appendix,* Fig. S7*D*).

Finally, to test whether endogenous synaptic acetylcholine release can induce a similar effect, we carried out optogenetic stimulation of cholinergic axons in a mouse line expressing channelrhodopsin-2-eYFP (ChR2-eYFP) under the control of the choline acetyltransferase (ChAT) promoter. CA3PCs in a wild type mouse line showed heterogeneity of Ca^2+^ spike kinetics, similar to (although less pronounced than) that in rat cells (*SI Appendix,* Fig. S7*E*). In ChAT-Cre/Ai32 mice we observed prominent eYFP^+^ axonal innervation in the perisomatic area in CA3 similar to other studies (33, 51–53) (Fig. 4*E*). Phasic photostimulation before and during somatic I_inj_ (33) increased the rate and duration of CSBs, and raised steady-state AP frequency at the same V_m_ compared to control stimulation without light (Fig. 4 *F* and *G*), whereas baseline V_m_ (*SI Appendix,* Fig. S7*F*) and basic AP properties (*SI Appendix,* Fig. S7 *G* and *H*) remained largely unaffected, except for a reduction of AHP during the steady-state phase of the current step (54) (*SI Appendix,* Fig. S7*H*).

Altogether these results reveal that CSB rate and duration are controlled by cholinergic activity in CA3PCs at least in part via modulating dendritic Ca^2+^ spike kinetics. As short Ca^2+^ spikes are expressed in a subpopulation of CA3PCs, increased cholinergic activity may specifically mediate a transition from regular spiking mode to firing long plateau potentials and complex spike bursts, potentially opening a state-dependent window for robust synaptic plasticity and rapid memory encoding by this CA3PC subtype (*SI Appendix,* Fig. S8).

## Discussion

Dendritic Ca^2+^ spikes have been observed in various neuron types since the advent of direct investigation of the electrophysiological properties of dendrites. Ca^2+^ spikes in PCs were typically described as stereotypical slow events lasting for tens of milliseconds (55), and have been considered to underlie the generation of CSBs (7, 10). While some reports indicated that pharmacologically isolated Ca^2+^ spikes could occur in complex forms composed of slow and fast components (8, 10, 56, 57), to our best knowledge these features have not been investigated systematically. However, recent reports demonstrated that, in certain PC types, Ca^2+^ spikes can occur in remarkably different forms (21, 22). In particular, an unusually fast (few ms long), dendritically initiated Ca^2+^ spike type has been described in apical trunk dendrites of human cortical layer 2/3 PCs (22) and of rat hippocampal CA3PCs (21). In rat CA3PCs, about half of the recorded higher-order apical dendrites expressed such fast Ca^2+^ spikes, whereas other dendrites mostly expressed slower Ca^2+^ spikes following backpropagating APs, and in some dendrites both types could be evoked (21). However, it remained unclear whether different dendrites in an individual neuron have typically uniform Ca^2+^ spikes (suggesting cell-level regulation of Ca^2+^ spikes), or variable Ca^2+^ spike properties (suggesting dendrite-level regulation). Evoking compound Ca^2+^ spikes by widespread dendritic depolarization from the soma, we found that many CA3PCs express combination of slow and fast Ca^2+^ spike components (e.g. complex cells in Fig. 1) suggesting possible heterogeneity among dendrites. However, in a large fraction of CA3PCs the compound Ca^2+^ spikes have very short duration (~10 ms), suggesting largely homogeneous fast Ca^2+^ spikes expressed in their dendrites. Alternatively, the heterogeneity of compound Ca^2+^ spike waveforms among cells could be explained by differences in voltage-sensitive ion channel properties and their dynamic interactions. Interestingly, the electrophysiological phenotype is associated with morpho-topographic cellular features: CA3PCs with short compound Ca^2+^ spikes are mostly located in proximal CA3 and superficial layers of distal CA3, have shorter and often multiple primary apical trunks, extensive dendritic arborization, and dense TEs. Under baseline conditions, these CA3PCs typically have low propensity to fire CSBs (19) and when they do, the CSBs are brief, consistent with their short-duration Ca^2+^ spikes.

Our results demonstrate that L-type VGCCs play the dominant role in generating both short- and long-duration Ca^2+^ spikes in CA3PCs. This is consistent with the relatively high voltage threshold of the spikes as well as with low expression of Ca_v_2.3 (mediating R-type VGCCs) in CA3PCs (58). ω-CTX MVIIC moderately reduced short Ca^2+^ spikes, indicating a small contribution of N-type VGCCs. It is still possible that distinct Ca_v_1 subunits or their cell subtype-dependent regulation contributes to the varieties of Ca^2+^ spikes (59). The strong correlation we found between dV/dt_max_ and dV/dt_min_ of Ca^2+^ spikes may suggest that higher VGCC conductance, leading to faster depolarization, could more effectively recruit the VGKCs producing the short spike form. However, this is unlikely because partial inhibition of the short Ca^2+^ spike (during the wash-in of L-type channel blockers or by ω-CTX MVIIC) or facilitation of the rate of rise of slow Ca^2+^ spikes (by K_Ca_ blockers) did not cause a switch in the spike form. Instead, our data suggest that differences in K_A_ and K_M_ currents (mediated by members of Kv4 and Kv7 channel families, respectively) are primarily responsible for the differences between the two main Ca^2+^ spike forms. In agreement with this, the Kv7.5 channel subunit is strongly expressed in CA3PCs (60), particularly in superficial cell layers (61), and Kv4.2 and Kv4.3 VGKC subunits are expressed in moderate to high densities in CA3PC dendrites (62). In contrast to the involvement of the above channels, to our surprise, we did not find a similar role for K_Ca_ channels, whose blockade paradoxically even facilitated fast repolarization of the short Ca^2+^ spike, perhaps due to an interaction with VGKCs (63). It will remain for future studies to dissect the cell subtype-specific molecular mechanisms regulating K^+^ channel activities, and possible dendritic morphological factors contributing to the electrophysiological differences observed here.

We found that cholinergic activation strongly prolongs the duration of short Ca^2+^ spikes, converting them into a long-lasting form. This effect — probably in concert with other ACh-mediated mechanisms (43, 64, 65) — allows sustained plateau-induced CSB firing, which is expected to efficiently induce synaptic plasticity (6, 16) and may provide a state-dependent dendritic mechanism contributing to memory encoding and retrieval. ACh is released dynamically on a time scale of seconds during explorative behavior and salient stimuli (66, 67), which often involve learning of new contexts and/or associations. A popular concept suggests that the wide range of cellular, synaptic, and network changes induced in CA3 by elevated ACh concentration act in concert to facilitate the encoding of novel information as opposed to retrieval of previously stored activity patterns in this attractor network (23). Cholinergic agonists increase bursting of CA3PCs (50, 68–70) in part due to changes in intrinsic electrical properties; yet, the specific effects of cholinergic receptor activation on active dendritic electrical properties of CA3PCs have not been experimentally directly investigated. In CA1PCs, cholinergic activation increases dendritic AP backpropagation and Ca^2+^ signaling (71, 72), and facilitates Ca^2+^ spikes due to inhibition of K^+^ conductances and enhancement of R-type VGCCs (46, 54, 73, 74). In cortical L5PCs, brief cholinergic stimulation also facilitates dendritic Ca^2+^ plateau generation, by augmenting R-type Ca^2+^ channels (25, 26). While this functional impact is similar to our current findings, R-type channels are unlikely to be the target of ACh in CA3PCs as these channels are weakly expressed (58) and according to our results do not substantially contribute to Ca^2+^ spikes in CA3PCs.

Along with other studies, our results support the emerging concept that CA3 is a complex network composed of morpho-functionally heterogeneous principal cells that differ in many properties, including their location, connectivity, morphology, dendritic properties, firing patterns, and state-dependent regulation, and that could perform specific computations (33, 75, 76). It is still unclear whether the diversity of CA3PCs represents a gradual spectrum of features or truly distinct subpopulations, and if the latter, how many groups exist. In juvenile mice and rats, Hunt et al distinguished two main subgroups called thorny and athorny cells, based on a combination of properties including morphology, burstiness, transcriptomics, and the presence or lack of TEs (33, 77). In our rat dataset apparently all cells possess TEs albeit in varying densities associated with different primary dendritic morphologies, similar to previous studies in adult rats (30). As another difference, cholinergic stimulation suppressed bursting in athorny cells (33) whereas we uniformly observed facilitation of CSBs. Further studies, involving genetic and immunohistochemistry tools are needed to conclusively define CA3PC subtypes and to clarify developmental and species-related differences. We nevertheless suggest distinction of two main morpho-functional CA3PC subtypes (with partially overlapping features, perhaps representing the two ends of the gradual spectrum) and speculate that they potentially serve different computational functions (*SI Appendix,* Fig. S8). One type of CA3PCs, located mostly in proximal CA3 and in the superficial layer of distal CA3, has low propensity for CSB firing under baseline conditions, associated with short Ca^2+^ spikes. High ACh levels enable these neurons to generate longer Ca^2+^ spikes and CSBs, and we hypothesize that only such ACh-gated long Ca^2+^ plateaus (but not short Ca^2+^ spikes) are able to efficiently induce rapid synaptic plasticity. These properties may suggest a primary involvement of these CA3PCs in encoding novel information during learning (74–77). First, most of these cells appear to receive abundant MF input, which fits with the proposed tight cooperation between proximal CA3c with DG to create novel representations during memory formation (78–81). Second, the increased dendritic excitability during cholinergic activity may enhance the capacity of MFs or distal entorhinal synaptic input (or their conjunctive action) to induce prolonged Ca^2+^ plateau potentials and plasticity of str. radiatum synapses, while presynaptic suppression of transmission at recurrent synapses would limit retrieval — and thereby interference with — previously encoded associations (23). The other subtype of CA3PCs is preferentially found relatively deeper in distal CA3; these cells are more heterogeneous but overall appear to receive less MF input, and they express CSBs with higher rate and longer duration due to long-lasting Ca^2+^ spikes even under low ACh condition. Since many of these cells express both fast and slow spike components, the potentially complex functions of these spikes, including their impact on input–output transformation and synaptic plasticity are difficult to predict. It will be crucial to determine whether different input types can activate different types or combinations of the spikes and how effective are short and long spikes to induce synaptic plasticity with and without cholinergic activity. We speculate that the variable Ca^2+^ spike properties may enrich the ability of these cells to balance between information storage and retrieval by engaging distinct forms of Ca^2+^ spikes (*SI Appendix,* Fig. S8). Yet, we acknowledge that our stimuli in the slice to mimic increased cholinergic activity differ from the physiological spatiotemporal activity of cholinergic axons; the modulation on CA3PC properties by ACh will ultimately have to be elucidated by in vivo experiments.

In conclusion, our study sheds light on the complex and heterogeneous nature of CA3PCs, and uncovers distinct, stereotypical forms of dendritic Ca^2+^ spikes and their modulation by cholinergic activation. Further research into the functional implications of this diversity and its relevance to memory processing, as well as the elucidation of the molecular mechanisms governing these properties, will be essential for advancing our understanding of hippocampal function and its role in cognitive processes.

## Materials and Methods

### Slice Preparation.

Adult (7 to 12-wk-old) male Wistar rats, male FVB/AntJ mice, and male and female ChAT-Cre/Ai32 transgenic mice were used to prepare acute slices from the middle to dorsal part of the hippocampus of both hemispheres (400-µm-thick transverse slices in rats and 300-µm-thick coronal or transverse slices in mice) as described (3, 19, 21), according to methods approved by the Animal Care and Use Committee of the Institute of Experimental Medicine, and in accordance with the Institutional Ethical Codex, Hungarian Act of Animal Care and Experimentation 40/2013 (II.14), and European Union guidelines (86/609/EEC/2 and 2010/63/EU Directives). Details of the procedure are described in *SI Appendix*.

To obtain expression of ChR2 in cholinergic fibers for optogenetic experiments, we crossbred ChAT-Cre (The Jackson Laboratory, RRID: IMSR_JAX:006410) and Ai32 (The Jackson Laboratory, RRID: IMSR_JAX:024109) mice.

### Patch-Clamp Recordings.

Slices were transferred to a custom-made submerged recording chamber under the microscope where experiments were performed at 32 to 34 °C in ACSF containing (in mM): NaCl 125, KCl 3, NaHCO_3_ 25, NaH_2_PO_4_ 1.25, CaCl_2_ 1.3, MgCl_2_ 1, glucose 25, Na-pyruvate 3, and ascorbic acid 1, saturated with 95 % O_2_ and 5 % CO_2_. In experiments using focal electrical stimulation of axons (*SI Appendix,* Fig. S6*G*) the CaCl_2_ concentration was raised to 2 mM to facilitate glutamate release. Cells were visualized using Zeiss Axio Examiner or Olympus BX-61 epifluorescent microscope under infrared illumination and water immersion lens (63× or 60× during recording, 20× or 10× for overview z-stacks, Zeiss or Olympus). In every slice, we selected a region within CA3 where the apical trunks of most neurons were oriented slightly downward to access neurons with mostly preserved dendritic arborization (directed into the tissue), and we targeted somata located >50 μm deep in the slice. Somatic patch pipettes (2 to 6 MΩ) were filled with a solution containing (in mM): K-gluconate 134, KCl 6, HEPES 10, NaCl 4, Mg2ATP 4, Tris2GTP 0.3, phosphocreatine 14 (pH = 7.25), typically complemented with 50 µM Alexa Fluor 594 and 100 µM Oregon Green BAPTA-1 (OGB-1) or Oregon Green BAPTA-6F (OGB-6F) (all fluorescent dyes from Invitrogen-Molecular Probes). Electrophysiological results were similar using OGB-1 and OGB-6F and therefore results obtained with different Ca^2+^-sensitive dyes were pooled.

After achieving GΩ seal resistance followed by patch membrane opening, current-clamp whole-cell recordings were performed using BVC-700 amplifiers (Dagan, Minneapolis, MN) in the active “bridge” mode, filtered at 3 kHz and digitized at 50 kHz. Series resistance was typically between 15 to 25 MΩ at the soma and 25 to 60 MΩ at the dendrite, frequently checked using small hyperpolarizing I_inj_ steps, and compensated with bridge balance and capacitance compensation; recordings were terminated if somatic series resistance exceeded 30 MΩ or if the membrane potential became unstable or persistently depolarized. Only CA3PCs with somatic resting membrane potential (V_m_) more negative than −60 mV after break-in were used for experiments. Cells were usually kept at −68 to −72 mV with appropriate constant current injection. After establishing the somatic whole-cell current clamp configuration, cells were loaded for >20 min to visualize the dendritic tree by 2P imaging. Application of carbachol usually led to a slow ~2 to 6 mV depolarization, which was compensated with hyperpolarizing current to keep V_m_ constant.

CSB rate (i.e. propensity) was measured using a series of somatic depolarizing current injections (five 100-ms-long steps of 300 to 600 pA with 80.55-ms-long interstep intervals) from ~−70 mV baseline V_m_, repeated 5 to 10 times with few second intervals. CSB duration was measured using 1-s-long I_inj_ steps at or slightly above the threshold level of current evoking CSBs.

The properties of Ca^2+^ spikes were determined after >10 min bath application of TTX, using 1-s-long step or ramp I_inj_. Typically a range of I_inj_ levels were tested with multiple repetitions, including subthreshold and different suprathreshold values that evoked the Ca^2+^ spike with different latencies. We aimed to set the I_inj_ to the level where the Ca^2+^ spike was evoked at the approximate middle of the step. In pharmacological experiments Ca^2+^ spikes were measured in repeated series 10 min apart under control conditions (to ensure that their properties were stable), and after application of the chemical for at least 10 min. Description and specificity of the chemicals used in this study is detailed in *SI Appendix*.

In all recorded CA3 neurons, dendrites were carefully inspected for thorny excrescences in proximal apical parent dendrites and small spines on more distal dendritic branches, and for verifying that no main proximal apical trunk was cut.

### Two-Photon Imaging and Uncaging.

Dual galvanometer based two-photon scanning systems (Bruker, former Prairie Technologies, Middleton, WI, USA) were used to image the patched neurons and to uncage glutamate at individual dendritic spines as previously described (19, 21). Two ultrafast pulsed laser beams (Chameleon Ultra II; Coherent, Auburn, CA) were used: one laser at 920 or 860 nm for imaging OGB dyes and Alexa Fluor 594, respectively, and the other laser tuned to 720 nm to photolyze MNI-caged-L-glutamate (Tocris). The intensity of the laser beam was controlled with electro-optical modulators (model 350-80, Conoptics, Danbury, CT). Linescan Ca^2+^ measurements were performed with 8 μs dwell time at ~200 to 300 Hz. Additional details on glutamate uncaging and Ca^2+^ imaging can be found in *SI Appendix*.

### Optogenetic Stimulation.

Acute slices were prepared from ChAT-Cre/Ai32 mice as described above. Cholinergic axons were activated using pulsed train illumination (100-ms pulses with 100-ms intervals, applied for 10 s (33) through the 60× objective with 447-nm laser light (Fig. 4*F*). While we cannot precisely mimic the firing dynamics of cholinergic axons projecting to the hippocampus, elevated acetylcholine release on a similar second-long time scale was observed in the hippocampus of awake behaving animals (66, 67). 1-s I_inj_ steps were applied either without preceding illumination (control) or during the end of the illumination train. CSB rate and duration were measured at the threshold I_inj_ level eliciting CSBs under control conditions (3 cells produced no CSBs with up to 1 nA I_inj_).

The effect of photostimulation on the baseline membrane potential (*SI Appendix,* Fig. S7*F*) was measured as the tenth percentile of data points in a 100-ms-long window, comparing V_m_ before (control) and during the photostimulation (before the I_inj_ step) on the very first trace with illumination.

### Data Analysis.

Analysis of voltage and Ca^2+^ recordings was performed using custom-written scripts in IgorPro (WaveMetrics, Lake Oswego, OR) and Python. Description of CSB analysis and Ca^2+^ spike parameter extraction and analysis can be found in *SI Appendix*.

Clustering of Ca^2+^ spikes measured in TTX was performed with the Ward hierarchical clustering method, using the sklearn.cluster module in Python. For each individual cell, the z-score normalized dV/dt_total_, log_10_(half-width), and number of peaks were considered.

Dendritic morphological and distance measurements were performed using ImageJ (NIH, Bethesda, MD) on stacked 2P images of dye-loaded neurons, collected at the end of the experiment. Additional details of the morphological analysis and STED can be found in *SI Appendix*.

### Statistical Analysis.

Statistical analysis was performed with the Statistica software (Statsoft, Tulsa, OK). Where possible, nonparametric tests (Wilcoxon test for two paired groups or for one sample compared to median=1, Mann–Whitney test for two unpaired groups, Kruskal–Wallis test with post hoc multiple comparisons for 3 unpaired groups, Friedman test for one-way repeated measures, Spearman correlation) were used, which do not make assumptions about the distribution of data. Mixed ANOVA with post hoc Tukey’s test was used for Sholl analysis. All statistical tests were two-tailed. Differences were considered significant when *P* < 0.05. In all figures, *P < 0.05; **P < 0.01; ***P < 0.001.

## Supplementary Material

Appendix 01 (PDF)

## Data Availability

All study data are included in the article and/or *SI Appendix*. The dataset is available in a public database (82).
