# Supplementary material for: Cholinergic regulation of dendritic Ca2+ spikes controls firing mode of hippocampal CA3 pyramidal neurons
Source: Proc Natl Acad Sci U S A. 2024 Nov 6;121(46):e2321501121. doi: 10.1073/pnas.2321501121 (PMC11572977; doi:10.1073/pnas.2321501121)
Supplement: Supplementary file 1 — Appendix 01 (PDF) [file pnas.2321501121.sapp.pdf]

PNAS

Supporting Information for

Cholinergic regulation of dendritic Ca^{2+} spikes controls firing mode of hippocampal CA3 pyramidal neurons

Noémi Kis[1,2], Balázs Lükő[1], Judit Herédi[3], Ádám Magó[1,4], Bela Erlinghagen[1], Mahboubeh Ahmadi[1,5], Snezana Raus Balind[1], Mátyás Irás[1], Balázs B. Ujfalussy[6], Judit K. Makara[1*]

Correspondence to: Judit K. Makara
Email: makara.judit@koki.hun-ren.hu

This PDF file includes:

Supplemental Methods

Figures S1 to S8

Supplemental References

Supplemental Methods

Slice preparation

Animals were deeply anesthetized with 5% isoflurane and quickly perfused through the heart with ice-cold cutting solution containing (in mM): sucrose 220, $NaHCO_3$ 28, KCl 2.5, NaH_2PO_4 1.25, $CaCl_2$ 0.5, $MgCl_2$ 7, glucose 7, Na-pyruvate 3, and ascorbic acid 1, saturated with 95 % O_2 and 5 % CO_2. The brain was quickly removed, and slices were prepared in cutting solution using a vibratome (VT1000S, Leica, Leica Biosystems GmbH, Nussloch, Germany). Slices were incubated in a submerged holding chamber in ACSF at 35 °C for 30 min and then stored in the same chamber at room temperature until use.

Electrophysiology

In some experiments (Fig. S6E), neurons in the ventromedial (VM) area of the thalamus were recorded in slices prepared with identical procedures from adult male calb1/ires2cre mice (50-80 days old) that were stereotaxically injected with the cre-dependent soluble mCherry expressing virus construct (AAV5-hSyn-DIO-mCherry) to identify the target area using 2P imaging.

In some experiments (Fig. S6G) focal electrical synaptic stimulation (two 0.1 ms pulses with 50 ms interval, BioStim stimulator system, Supertech Ltd, Pécs, Hungary) was performed using silver wires inserted into a theta pipette filled with ACSF containing 2 mM $CaCl_2$.

Dual soma-dendrite recordings were performed as previously described (1). The dendrite was patched first (pipette resistance 6–10 MΩ), and after establishing the dendritic whole-cell configuration, cells were loaded for >10 minutes to visualize the soma for targeted patching. Intracellular solution and recording settings were the same at the dendrite as at the soma. Other electrophysiological properties analyzed from the dual experiments included in the current study have been presented previously (1).

Two-photon glutamate uncaging

MNI-caged-L-glutamate (Tocris; 10 mM in ACSF) was applied through a puffer pipette with a ~20-30-μm-diameter, downward-tilted aperture above the slice using pneumatic ejection system (PDES-02TX, NPI, Tamm, Germany). Glutamate uncaging (Fig. S7B) was performed at a clustered set of 20 spines on a higher order apical trunk at 211 ± 26 μm distance from the soma (mean ± SEM, n=9 experiments), using 0.5 ms uncaging duration at each spine with 0.1 ms intervals between synapses, repeated 5 times at 40 Hz (1, 2). The experiments were carried out in the presence of 50 μM D-AP5 to block NMDA receptors. Uncaging laser power was adjusted to evoke summated voltage responses slightly above and below the threshold of APs, so that both subthreshold and moderately suprathreshold (AP evoked by any of the last three of the five stimuli) responses could be investigated.

Analysis of Ca^{2+} signals

Ca^{2+} signals are expressed as $\Delta F/F_0 = (F(t)-F_0)/F_0$, where F(t) is fluorescence at a given time point and F_0 is the mean fluorescence during 50 ms preceding the depolarizing I_{inj}. To measure Ca^{2+} spike associated Ca^{2+} signal amplitude, traces were aligned to the initial rise (~300 mV/s dV/dt value) of the Ca^{2+} spike, and we calculated the difference between the maximum average of 5 consecutive points following the spike and the average of the 20-50 ms period preceding the spike. Ca^{2+} traces and some of the electrophysiological traces are presented with slight smoothing (binomial, num=1).

Analysis of CSBs and Ca^{2+} spike properties

CSBs were identified similarly to that described (2) based on the combination of the following properties: (1) ≥ 2 high-frequency APs with progressively decreasing peak amplitude, preceded by at least one additional simple AP in the given I_{inj} pulse, (2) riding on an underlying slow afterdepolarization that followed the first AP of the CSB, and (3) typically accompanied by a Ca^{2+} signal in an apical dendrite. CSB rate in Fig. 1I and SI Appendix Fig. S3A-B was calculated by dividing the number of I_{inj} steps displaying CSB with the total number of I_{inj} steps (5 per trace, 5-10 repetitions). Recorded neurons were sorted into two groups: CSB producing cells (CSB cell; CSBs at I_{inj} threshold ≤600 pA) and regular spiking cells (RS cell; no CSB or CSB I_{inj} threshold >600 pA). The duration of full-blown CSBs evoked by I_{inj} was measured using the 1-second-long I_{inj} steps at or slightly above the lowest current evoking CSBs. CSB duration was calculated as the time difference between the peak of the last simple ('reference') AP before the CSB and the peak of the last AP either (i) with reduced amplitude compared to reference AP or (ii) fired <50 ms after the preceding AP, whichever comes first. AP threshold was measured as the voltage value where dV/dt exceeded 20 V/s.

To analyze Ca^{2+} spike properties measured in TTX at the soma (illustrated in Fig. S1E), traces were smoothed using the built-in binomial (Gaussian) smoothing algorithm (num=10000) of IgorPro. dV/dt_{max} and dV/dt_{min} were determined as the maximum and minimum of the derivative of this smoothed trace, respectively. We defined the parameter dV/d_{total} as the full dV/dt range (dV/dt_{max} minus dV/dt_{min}). Spike threshold was measured as the voltage where dV/dt crossed an adaptive threshold (20% of the maximum of the first spike-associated dV/dt peak with an upper bound of 0.25 V/s). Amplitude was measured as voltage peak minus threshold; halfwidth was calculated as the time difference at half maximum amplitude. We note that the smoothing procedure slightly reduced the amplitude and increased the halfwidth of the short Ca^{2+} spikes (by maximum ~12%), but these effects do not affect our qualitative conclusions. The number of peaks was calculated using a custom Python algorithm where we take all the local maxima and discard those which are not separated by a local minimum that is at least 3 mV smaller than maxima on either side. The above parameters were measured on multiple (typically 5) repetitions and averaged to represent Ca^{2+} spike properties of a cell. In some of the experiments testing the effect of carbachol on Ca^{2+} spikes, the slow repolarization after the Ca^{2+} spike prevented the precise measurement of the halfwidth; in these cases we considered halfwidth to be 0.3 s.

In a small subset of CA3PCs, the step I_{inj} invariably evoked the Ca^{2+} spikes at the beginning of the step (<100 ms) where kinetic parameters could not be adequately measured. These cells were either excluded from detailed Ca^{2+} spike analysis, or alternatively we applied a ramp protocol using linearly increasing current for 1 second (Fig. S1A) that allowed extraction of the parameters.

The analysis of Ca^{2+} spike properties evoked at the soma in dual soma-dendrite recordings (Fig. S2) was performed as described previously (1).

Morphological analysis

Alexa Fluor 594 fluorescence was used for morphological analysis. The analysis was done blind to the electrophysiological cluster identity of the cells. Primary apical trunk length was measured on the 2D z-stacks by manually drawing a segmented line from the apical edge of the soma to the first main bifurcation. A cell was considered to have a single primary trunk if the initial thick apical trunk was at least 10 μm long before the first branchpoint. In cells with multiple primary trunks the length of all primary trunks was averaged as a measure of primary trunk length. Radial soma position was determined for a subset of cells where 2P Dodt contrast images were also taken,

and was measured as the Euclidean distance of the soma center from the border of str. pyramidale and str. lucidum.

To characterize the complexity of the apical dendritic arbor, Sholl analysis was performed on the 2D maximal intensity projections of the z-stacks in a subset of the recorded CA3PCs, in which the apical dendritic arborization was fully contained and well visualized in the 2P z-stack until at least 200 μm apical distance from the soma. We measured the number of apical dendritic intersections along concentric circles with 50, 100, 150 and 200 μm radius, centered at the soma.

The relative proximodistal position was calculated based on measurements on low-magnification fluorescent images of the whole hippocampus, as described (2). The distal border of CA3a (from CA2) was considered to be located at 200 μm from the sudden widening of the pyramidal cell layer (i.e., CA1–CA2 border). For separate analysis of proximal (relative position 0-0.3) and distal (relative position: 0.4-1) CA3PCs in Fig. 2B, cells in the transition zone (relative position between 0.3-0.4) were not included. Proximal CA3 approximately corresponds to CA3c, and distal CA3 roughly corresponds to CA3a and CA3b (2, 3).

We employed logistic regression to predict the binary class (short- versus long-duration Ca^{2+} spike) of the neurons based on either anatomical properties. We used either topographic (soma radial depth and proximo-distal position) or morphological features (number and average length of trunks, and the number of intersections from Sholl analysis at 50, 100, 150 and 200 μm distances), or both, as predictors. In this analysis we only included neurons where all of the above data were available (n=132 cells). Data reported are mean ± SD over 10 batches obtained from 10-fold cross-validation. In each batch, we used a different set of cells randomly subsampled from the whole population to test the accuracy of a classifier trained on the remaining neurons.

Immunohistochemistry

To amplify ChR2-eYFP signal in slices from ChAT-Cre/Ai32 mice, tissue samples were incubated with rabbit anti-GFP primary antibody (1:1000, Millipore) overnight and with anti-rabbit IgGs coupled with Cy3 for 2 hours (1:500, Jackson ImmunoResearch) in TBS containing 0.2% Triton-X100. Fluorescent images of ChR2-eYFP expression were taken using a Nikon Eclipse Ci-L Plus microscope with a 10x objective.

STED

Recorded neurons in the deep layer of distal CA3 were filled for ~20 min using regular pipette solution complemented with 7 mM biocytin. After careful withdrawal of the patch pipette, slices were placed in a fixative containing 4% formaldehyde and 0.2% picric acid in 0.1 M phosphate buffer (PB) (pH = 7.4) overnight at 4 °C. Then until further anatomical processing they were kept in PB containing 0,05 % sodium azide at 4 °C. To resection tissue samples, they were embedded in agarose (2%) and cut at ~100 µm thickness using a Leica vibratome (VT1000S). To visualize biocytin-filled cells, slices were incubated with A635P-conjugated streptavidin (1:1000, Abberior) in TBS containing 0.2% Triton X-100 overnight at RT. Biocytin-filled cells and thorny excrescences were imaged using a confocal Olympus FV3000-BX63 and an Abberior Instruments Expert Line STED microscope.

Chemicals

Tetrodotoxin (Tocris or Alomone Labs), guangxitoxin, XE991 dihydrochloride, mibefradil dihydrochloride, NNC55-0396 dihydrochloride, iberiotoxin, apamin, carbachol, hexamethonium bromide, D-AP5 (Tocris), SNX-482 (Peptides International), ω-Conotoxin MVIIC, dendrotoxin-I, AmmTx3 (Alomone Labs) and ipratropium bromide (Sigma-Aldrich) were prepared in stock

solution in distilled water. For peptide toxins the solvent also contained 0.1% BSA (Sigma-Aldrich). Nimodipine, nifedipine (Tocris) and TTA-P2 (Alomone Labs) were dissolved in DMSO. Stock solutions were stored at –20°C and dissolved to final concentration (in at least 1:1000 dilution) into bubbled ACSF before application. In experiments with peptide toxins, the perfusion tubes were pretreated with 0.1% BSA (dissolved in ACSF) to prevent the toxin from binding to the wall.

Tetrodotoxin at 1 μM concentration is widely used to block VGNCs (4, 5) and it completely eliminated APs in our experiments. As an independent confirmation that Ca^{2+} spikes in TTX are not mediated by incompletely blocked VGNCs, replacement of NaCl with equal concentration of NMDGCl (to replace the majority of extracellular Na^+ ions with the large cation $NMDG^+$ which permeates less through VGNCs) in the presence of 1 μM TTX produced no change in Ca^{2+} spike properties (Fig. S1F).

Ca^{2+} channel blockers at the concentration applied in this study are generally considered selective to the targeted Ca^{2+} channel subtype. TTA-P2, NNC55-0396 and mibefradil inhibit primarily T-type calcium channels (6-10) although mibefradil is relatively less selective; SNX-482 specifically blocks R-type Ca^{2+} channels (11); ω-Conotoxin MVIIC inhibits N-type and P/Q-type Ca^{2+} channels (12); and nimodipine and nifedipine are antagonists of L-type Ca^{2+} channels (13-14). We note that SNX-482 has been reported to also inhibit Kv4 potassium channels (15), however we did not observe an effect of SNX-482 on the halfwidth of Ca^{2+} spikes, arguing against a confounding effect on K^+ channels.

The concentration of carbachol we applied (2 µM) is expected to activate acetylcholine receptors in the physiological range: the concentration of acetylcholine during awake exploration or memory tasks has been estimated to rise to the ~0.1-1 µM range (16-20), and CCh has a several-fold lower affinity for mAChRs compared to acetylcholine (21-22).

Supplemental Figures

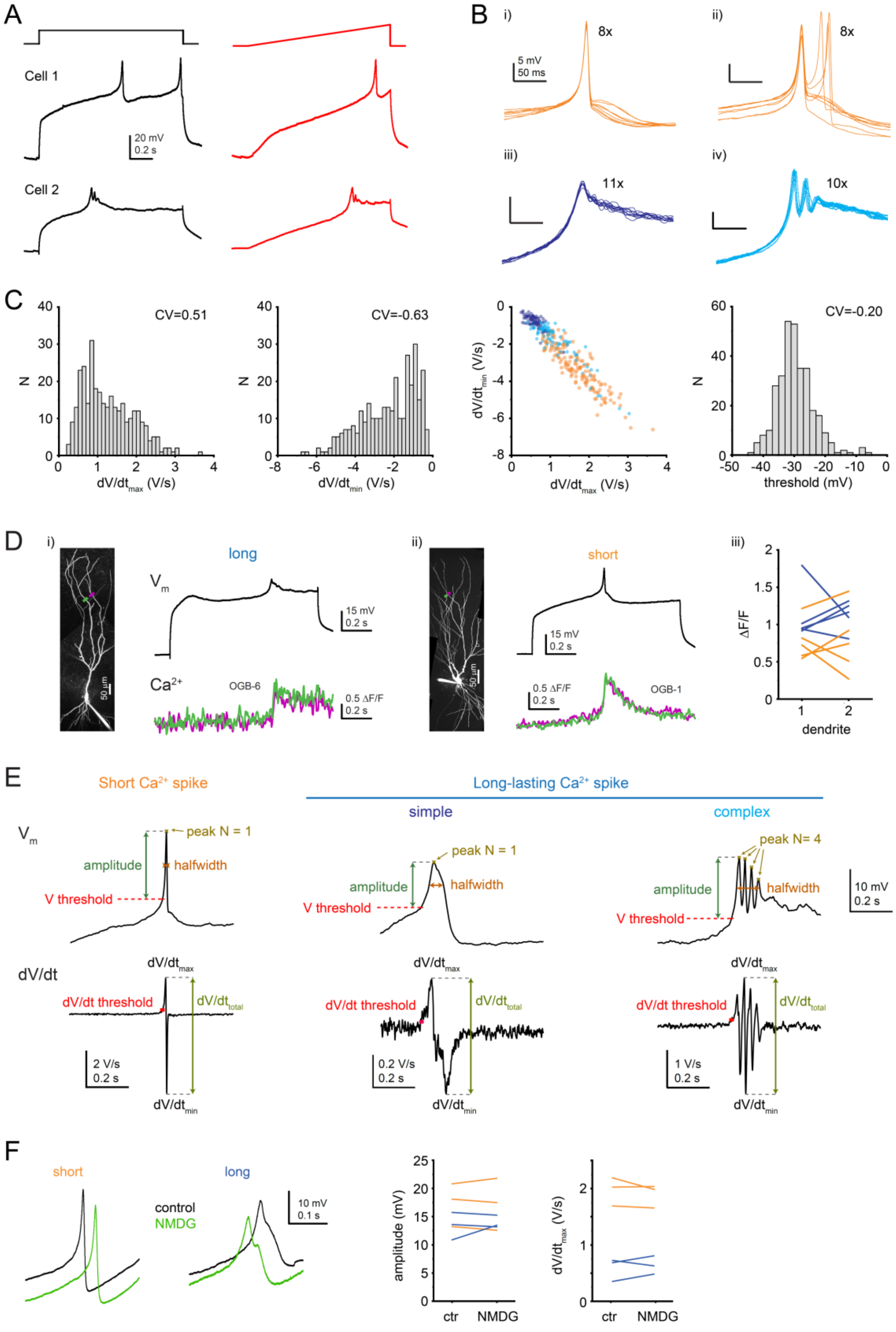

Figure S1. Classification and properties of Ca^{2+} spikes in TTX

A) 1-second-long step (black) and ramp (red) current injection in two different CA3PCs. Note the similar Ca^{2+} spike profiles using the two different depolarization protocols.

B) Example cells showing multiple repetitions of Ca^{2+} spikes with short (i-ii) or long (iii-iv) duration aligned to the first peak. Note that Ca^{2+} spike waveforms are usually highly stereotypical (i, iii, iv). In some cases of short Ca^{2+} spikes (ii), occasionally a second peak with variable jitter was also evoked.

C) Non-Gaussian distribution of different Ca^{2+} spike parameters in the recorded cell population (Shapiro-Wilks test for normality: dV/dt_{max}: $p<0.001$; dV/dt_{min}: $p<0.001$; threshold: $p<0.001$; n=317), and the strong inverse correlation between dV/dt_{max} and dV/dt_{min} (Spearman R=-0.94, $p<0.001$).

D) Example experiments in CA3PCs with long (i) and short (ii) Ca^{2+} spikes, in which Ca^{2+} signals were measured simultaneously in two distal dendritic segments belonging to different apical dendritic subtrees. Left, 2P z-stack of a CA3PC; green and purple lines indicate linescan locations in the two dendrites. Right, representative Ca^{2+} spike (V_m) and corresponding dendritic Ca^{2+} responses. iii) Summary of the paired dendritic Ca^{2+} signals (averaged from 5 repetitions) in cells with long (n=5 cells, blue) and short (n=5 cells, orange) Ca^{2+} spikes.

E) Definition of compound Ca^{2+} spike parameters. Top: example somatic voltage responses to I_{inj}; bottom: the corresponding dV/dt belonging to the Ca^{2+} spike. Note that the same scale bar applies for all V_m traces, whereas the Y scales of dV/dt traces are different.

F) Left, example recordings of short- and long-duration Ca^{2+} spikes under control conditions (black; normal ACSF containing 1 μM TTX) and after replacement of NaCl with NMDG-Cl in the extracellular solution (red; 1 μM TTX continued). Right, Summary of the impact of replacing Na^+ by $NMDG^+$ on short- (n=3, orange) and long-duration (n=3, blue) Ca^{2+} spike amplitude and dV/dt_{max}.

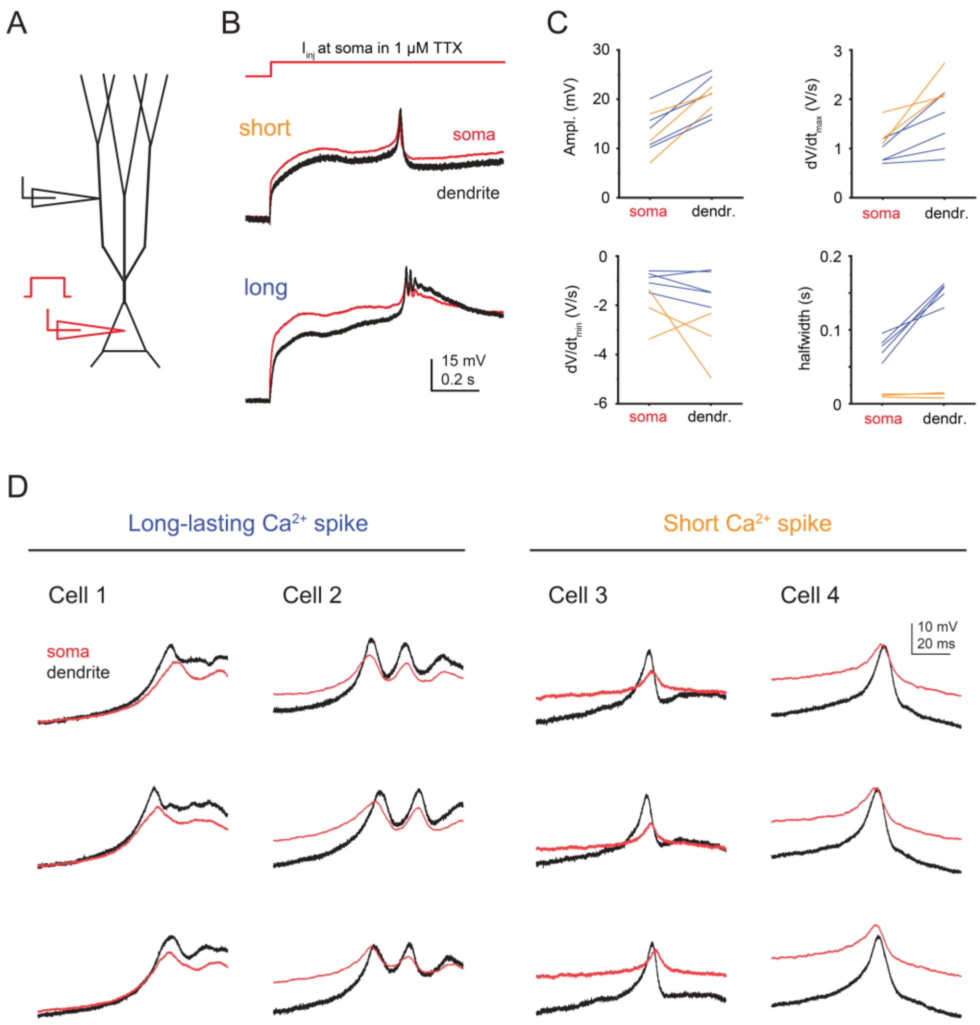

Figure S2. Dual soma-dendrite recordings in CA3PCs

A) Schematic of dual recordings from the soma and a higher-order apical dendrite.

B) Example soma-dendrite dual recordings with short and long Ca^{2+} spikes evoked by somatic I_{inj} (top) in 1 μM TTX. Note the close correspondence of the Ca^{2+} spike type in the dendrite and the soma.

C) Comparison of various Ca^{2+} spike parameters measured in the dendrite and at the soma, for short (orange, n=3 experiments) and long-lasting (blue, n=5 experiments) Ca^{2+} spikes. Results of Wilcoxon tests for the pooled data (n=8): amplitude: p=0.011; dV/dt_{max}: p=0.011; dV/dt_{min}: p=0.161; halfwidth: p=0.025.

D) Rising phase of Ca^{2+} spikes in example dual recordings with long-lasting (left) and short (right) Ca^{2+} spikes. Note that the Ca^{2+} spikes typically rose slowly without a clear threshold from a baseline that sometimes had different dynamics at the dendritic and somatic recording sites, altogether making the definition of spike onset time too uncertain for the assessment of relative timing. In addition, the peak of the spikes showed different relative timing between the somatic and dendritic recording site across different cells (compare Cell 1 vs 2 or Cell 3 vs 4) and occasionally also varied across trials within a cell (e.g. Cell 1).

Supplemental discussion on the site of Ca^{2+} spike initiation

The results of the dual recordings (Fig. S2) and the presence of tightly spike-associated all-or-none Ca^{2+} signals even in the most distal apical dendrites (Fig. 1D, 1F, S1D) collectively demonstrate that the somatic depolarization efficiently recruited regenerative Ca^{2+} spikes in the apical dendrites. Nevertheless, we emphasize that our experiments do not conclusively indicate where the Ca^{2+} spikes were initiated.

Several considerations complicate the determination of Ca^{2+} spike initiation site. The slow rise of Ca^{2+} spikes lacking a distinct threshold makes the assessment of relative timing of Ca^{2+} spike onset in different compartments unreliable, similarly to that concluded in a previous study in CA1PCs (23). Furthermore, under our stimulation conditions (depolarization at the soma in TTX) it is unlikely that the Ca^{2+} spikes are initiated at a single well-defined location. In addition, in case of dendritic initiation, the extensive branching of the apical dendritic arbor of CA3PCs may allow Ca^{2+} spikes to be generated in one or few dendritic branches and propagate to the rest of the dendritic arbor (e.g. we previously speculated that slow spikes may be generated in the main apical trunk (1)). In such cases, the timing of the event at two recording sites can be expected to depend on the actual position of the recording locations relative to the Ca^{2+} spike initiation site(s).

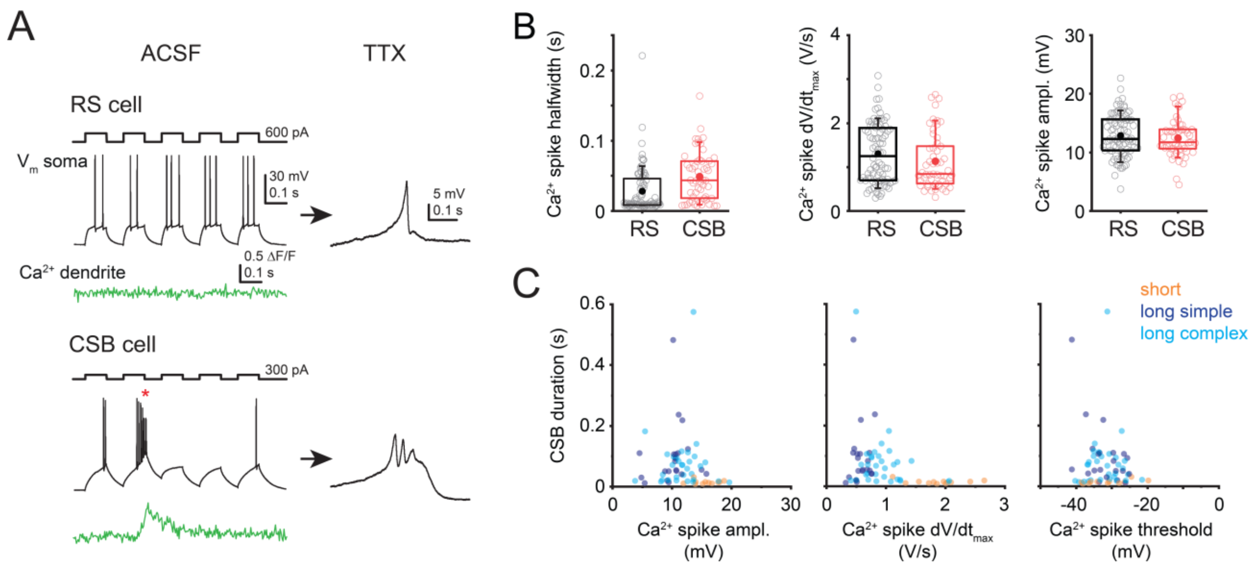

Figure S3. Relationship of CSBs and Ca^{2+} spike properties

A) Left, representative voltage (black) and distal dendritic Ca^{2+} responses (green) to repetitive 100-ms somatic I_{inj} in ACSF in a RS cell (top, 600 pA I_{inj}) and a CSB cell (bottom, 300 pA I_{inj}). Right, Ca^{2+} spike measured after the application of TTX using 1-second-long I_{inj} (segments cut from 1-second-long I_{inj} steps).

B) Ca^{2+} spike properties in TTX in RS (black, n=84) and CSB (red, n=53) cells. Mann-Whitney test revealed difference in Ca^{2+} spike halfwidth ($p<0.001$) but not in dV/dt_{max} ($p=0.174$) or amplitude ($p=0.494$). Open circles: individual cells; box: interquartile interval; line: median; filled circle: mean; whiskers: 10-90%.

C) CSB duration (measured using 1-second-long I_{inj} steps) showed weak negative correlation with Ca^{2+} spike amplitude (Spearman R=-0.253, p=0.044, n=63), strong negative correlation with dV/dt_{max} (Spearman R=-0.439, $p<0.001$) and no correlation with threshold (Spearman R=-0.028, p=0.824). Note that the analyses of CSB duration vs. Ca^{2+} spike properties included data recorded from cells that were categorized as RS cells (no CSB by repetitive 100-ms I_{inj} at ≤600 pA, see protocol in A and Methods) but that were able to fire brief CSBs at high levels of I_{inj}. This is consistent with our previous work (2) showing that several RS cells are able to express CSBs when somatic 600 pA I_{inj} is combined with synaptic stimulation in distal apical dendrites.

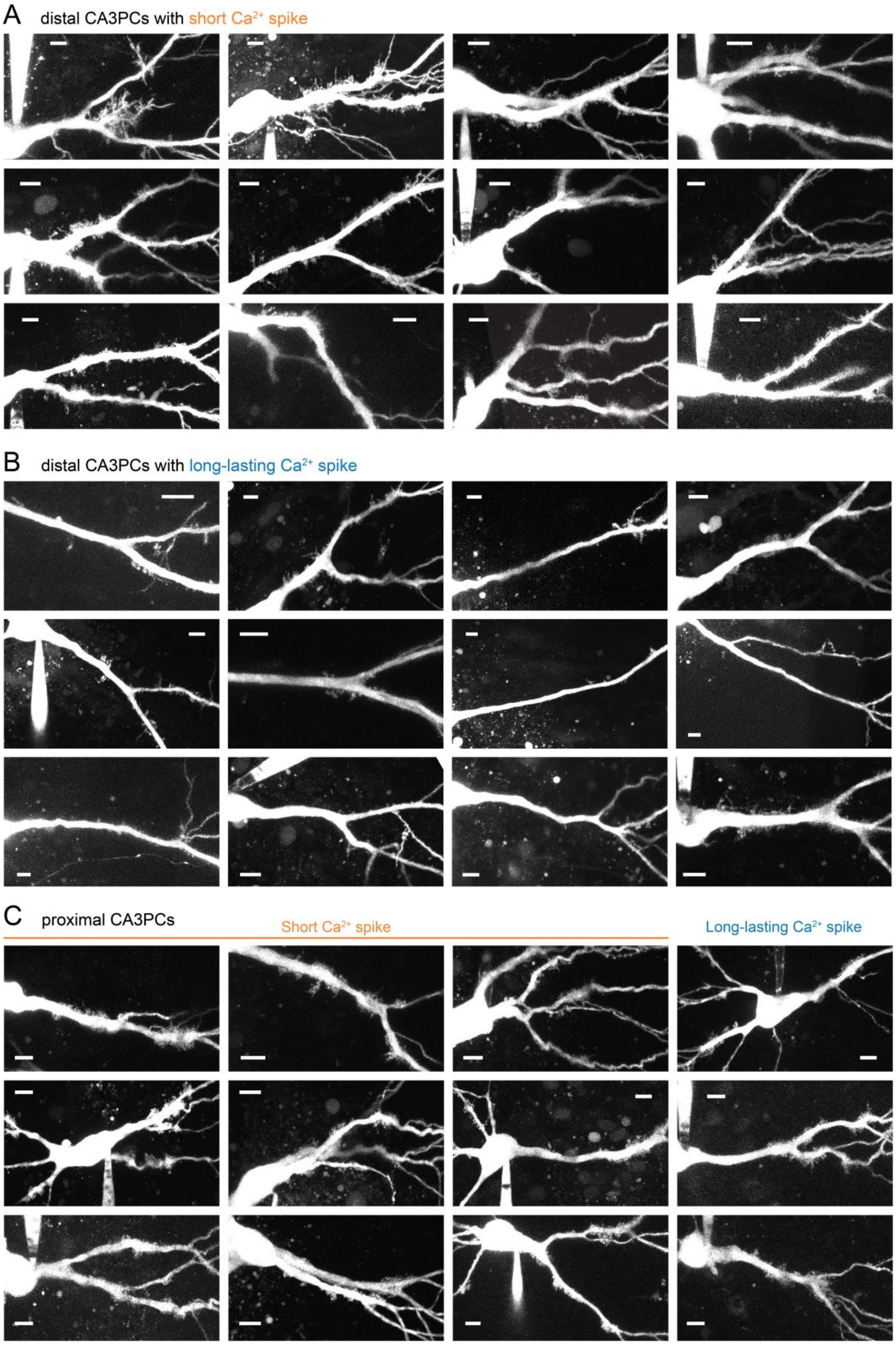
A
distal CA3PCs with short Ca2+ spike
B
distal CA3PCs with long-lasting Ca2+ spike
C
proximal CA3PCs
Short Ca2+ spike
Long-lasting Ca2+ spike

Figure S4. Collection of two-photon z-stack maximal intensity projection images of the proximal apical dendrites of distal CA3PCs.

A) Example PCs expressing short Ca^{2+} spikes in distal CA3. Note the early branching and abundant moss-like thorny excrescences (TEs) on the apical trunks.

B) Example PCs expressing long-lasting Ca^{2+} spikes in distal CA3. The cells also had TEs but typically with a lower density and often on relatively distal segments of the apical trunk. See also STED imaging of similar cells in Fig. 2I-N.

C) Example PCs from the proximal CA3 with short and long-duration Ca^{2+} spikes, heavily decorated with TEs on the trunk and occasionally also on basal dendrites.

Scale bars in A-C: 10 μm.

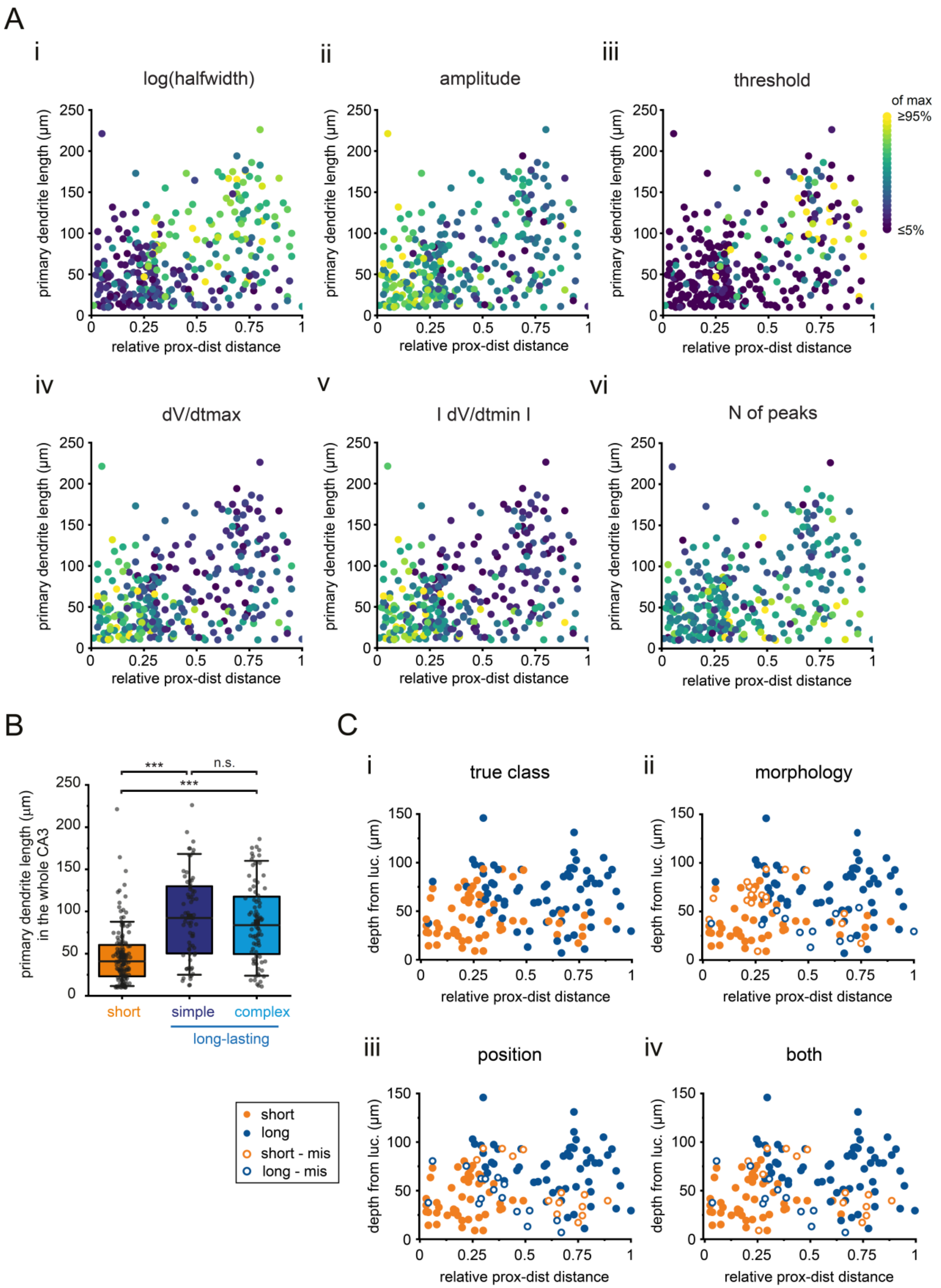
A
i
log(halfwidth)
ii
amplitude
iii
threshold
of max
≥95%
≤5%
iv
dV/dtmax
v
I dV/dtmin I
vi
N of peaks
primary dendrite length (µm)
relative prox-dist distance
B
primary dendrite length (µm) in the whole CA3
***
n.s.
short
simple
complex
long-lasting
C
i
true class
ii
morphology
iii
position
iv
both
depth from luc. (µm)
short
long
short - mis
long - mis

Figure S5. Further analysis of the relationship between Ca^{2+} spikes properties and cell position and morphology

A) Distribution of various Ca^{2+} spike parameters depending on proximodistal position and apical trunk length of CA3PCs. Each dot represents an individual cell (n=299), with colors indicating the relative magnitude of the kinetic parameters of the spikes: i) $\log_{10}$(halfwidth) [7.1 - 112 ms], ii) spikes amplitude [5.7 - 19.7 mV], iii) threshold [-40.5 - -17.3 mV], iv) dV/dtmax [0.38 - 2.7 V/s], v) the absolute value of dV/dtmin [0.46 - 5.2 V/s], vi) average number of peaks [1 - 3]. The relative proximodistal position is measured from dentate gyrus (0) to the border of the CA2 region (1; see Methods). See also Fig. 1H for the distribution of the kinetic parameters.

B) Difference in primary apical dendrite length between CA3PCs with short and long-lasting Ca^{2+} spikes in the whole CA3 region. Kruskal-Wallis test: $p<0.001$; post hoc multiple comparisons: short vs simple long: $p<0.001$, short vs complex long: $p<0.001$, simple long vs complex long: $p=1$.

C) Classification of Ca^{2+} spike types based on morpho-topographic features. Here we used only a subset of the neurons (n=132) where both all topographic parameters (proximodistal position and radial depth) and morphological analysis (Sholl analysis) were available.

i) The primary class of the neurons used in this analysis (colors, based on the clustering of the Ca^{2+} spikes shown in Fig 1G-H) as a function of their proximodistal and radial position.

ii) Cross-validated class prediction based on morphological features (number of trunks, average length of trunks and number of intersections at 50, 100, 150 and 200 μm) using logistic regression. Color indicates the true class labels, empty circles show misclassified neurons.

iii) Cross-validated class prediction based on topographic (proximodistal and radial) position.

iv) Cross-validated class prediction based on both morphological features and topographic position.

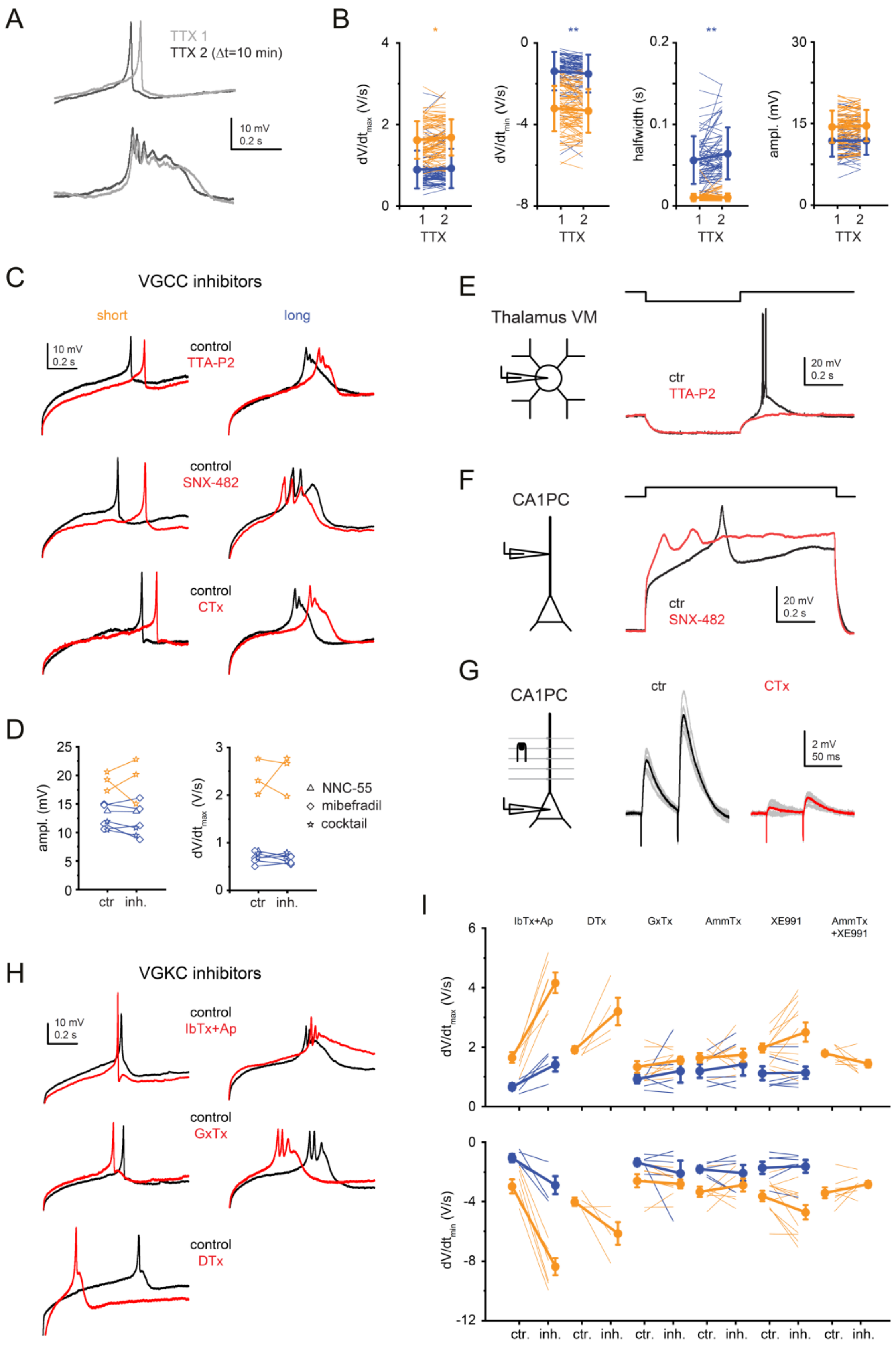

A
TTX 1
TTX 2 (Δt=10 min)
10 mV
0.2 s
B
dV/dtmax (V/s)
dV/dtmin (V/s)
halfwidth (s)
ampl. (mV)
TTX
C
VGCC inhibitors
short
long
control
TTA-P2
SNX-482
CTx
D
ampl. (mV)
dV/dtmax (V/s)
NNC-55
mibefradil
cocktail
ctr
inh.
E
Thalamus VM
ctr
TTA-P2
20 mV
0.2 s
F
CA1PC
SNX-482
G
CTx
2 mV
50 ms
H
VGKC inhibitors
IbTx+Ap
GxTx
DTx
I
IbTx+Ap
DTx
GxTx
AmmTx
XE991
AmmTx
+XE991
dV/dtmax (V/s)
dV/dtmin (V/s)
ctr.
inh.

Figure S6. Pharmacological characterization of Ca^{2+} spike forms

A) Examples of short (top) and long-lasting (bottom) Ca^{2+} spikes in TTX recorded 10 minutes apart before the application of an ion channel inhibitor.

B) Summary of the kinetic parameters of short (orange, n=82 cells) and long-lasting (blue, n=70 cells) Ca^{2+} spikes measured 10 minutes apart in TTX before the application of various ion channel inhibitors. *p<0.05, **p<0.01, Wilcoxon test. Note that even statistically significant differences in the large sample had small effect sizes, and cannot explain the main effects of VGCC and VGKC blockers.

C) Representative recordings of short (left) and long-lasting (right) Ca^{2+} spikes using the T-type VGCC inhibitor TTA-P2 (10 μM, top), R-type VGCC inhibitor SNX-482 (0.5 μM, middle) or the N/P/Q-type VGCC inhibitor ω-conotoxin-MVIIC (1 μM, bottom).

D) Summary of the effect of alternative T-type VGCC inhibitors (20 μM NNC-55; 10 μM mibefradil; cocktail of 10 μM TTA-P2, 10 μM mibefradil and 20 μM NNX–55) on the amplitude (left) and dV/dt_{max} (right) of short (orange) and long-lasting (blue) Ca^{2+} spikes. Each line represents an individual experiment.

E-G) Positive control experiments for SNX-482, TTA-P2 and ω-conotoxin MVIIC.

E) TTA-P2 (10 μM) eliminated low threshold spikes (LTS) in neurons in the ventromedial thalamus, in accordance with the established role of T-type VGCCs in these events. LTS were triggered after the end of -200 pA, 200-500 ms long hyperpolarizing I_{inj} steps from ~-65 mV resting V_m. Representative of 3 experiments with similar results.

F) SNX-482 (0.5 μM) blocked a substantial component of dendritic Ca^{2+} spikes in CA1PCs (tested in two experiments), in accordance with the established role of R-type VGCCs in these events. Ca^{2+} spikes were evoked with direct I_{inj} to the main apical trunk of CA1PCs (patched >200 μm from the soma) in the presence of 1 μM TTX.

G) ω-conotoxin MVIIC (1 μM) inhibited synaptically evoked EPSPs in CA1PCs, in accordance with the role of presynaptic P/Q- and N-type VGCCs in glutamate release from Schaffer collaterals. EPSPs were evoked with paired focal stimulation (50 ms interval) of Schaffer collaterals using a theta pipette with gently broken tip placed in str. radiatum, and recorded at the soma of CA1PCs in ACSF. Representative of 3 experiments with similar results.

H) Representative recordings of short (left) and long-lasting (right) Ca^{2+} spikes using (top) a combination of the BK channel inhibitor IbTx (0.1 μM) and SK channel inhibitor apamin (0.1 μM), (middle) the Kv2 VGKC inhibitor GxTx (0.1 μM) or (bottom) the Kv1 VGKC inhibitor DTX (0.1 μM).

I) Summary of the effect of various VGKC type inhibitors on the dV/dt_{max} (top) and dV/dt_{min} (bottom) of short (orange) and long-lasting (blue) Ca^{2+} spikes.

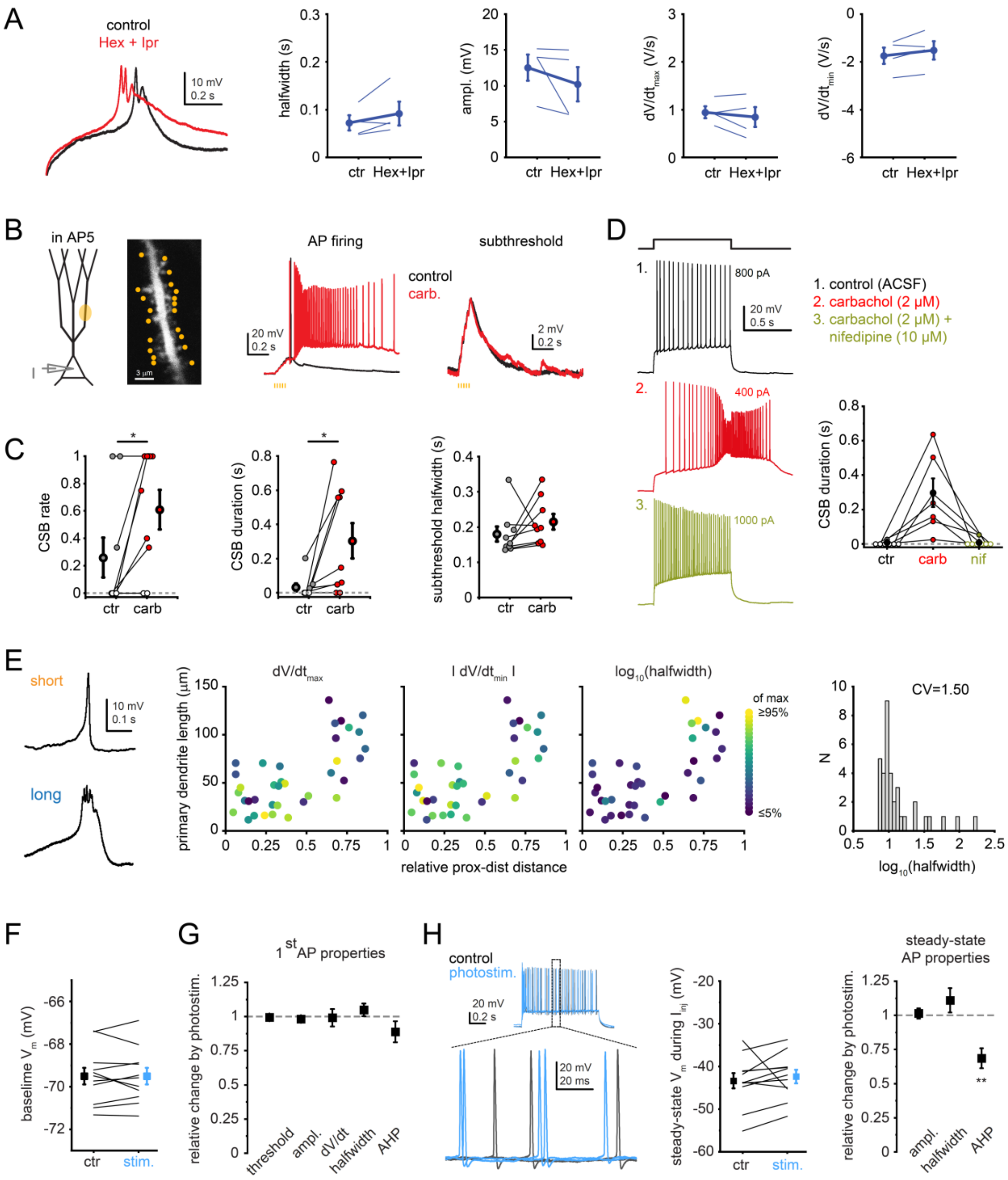

Figure S7. Additional results related to the cholinergic regulation of Ca^{2+} spikes

A) Left, example of long-lasting Ca^{2+} spike in TTX (control, black) and after application of 100 µM hexamethonium and 10 µM ipratropium (red). Right, summary of the effect of hexamethonium and ipratropium on long-lasting Ca^{2+} spike properties.

B) Effect of carbachol on somatic firing pattern evoked by synaptic stimulation. Left, schematic of the experiment, indicating the typical location of two-photon glutamate uncaging. Middle,

single frame scan of a higher-order apical trunk indicating the 20 synapses stimulated by 2PGU (yellow dots). Right, example suprathreshold and subthreshold voltage responses to 2PGU (20 spines stimulated quasi-synchronously 5x@40 Hz) under control conditions and in the presence of carbachol (2 μM). To avoid activation of confounding NMDA spikes, these experiments were performed in the continuous presence of an NMDAR blocker in the bath (D-AP5, 50 μM).

C) Summary of the effect of carbachol on CSB rate (left), CSB duration (middle) and the halfwidth of subthreshold responses with comparable amplitudes (right) in response to 2PGU stimulation (n=9 cells). White dots: no CSB; grey dots: CSB in control; red dots: CSB in carbachol. *p<0.05, Wilcoxon test.

D) Left, representative traces from a CA3PC stimulated with I_{inj} in ACSF (black), after 2 μM carbachol application (red) and after consecutive application of 10 μM nifedipine with carbachol (green). Note the prolonged CSB (outlasting the I_{inj} step) in carbachol, which is eliminated by nifedipine even at larger I_{inj}. Right, summary of CSB duration with carbachol and carbachol plus nifedipine (n=7 cells). White circles: no CSB; colored circles: with CSB. Filled black circles and error bars represent mean ± SEM.

E) Different types of Ca^{2+} spikes in mice. Left, example short and long-lasting Ca^{2+} spike evoked by I_{inj} in two CA3PCs from wild type FVB/AntJ mice. Middle, distribution of various Ca^{2+} spike parameters (color-coded based on the parameters indicated on top) depending on proximodistal position and apical trunk length of mouse CA3PCs. Right, histogram of the $\log_{10}$(halfwidth) values. Note the similarity to the heterogeneous distribution in rat CA3PCs in Figure 1E.

F) Baseline membrane potential measured before (ctr) and during (stim, blue) optogenetic cholinergic stimulation immediately before I_{inj}. Lines: individual neurons from n=11 experiments, symbols: mean ± SEM. Wilcoxon test, p=0.929.

G) Relative change in kinetic properties of the first AP by photostimulation of cholinergic axons in ChAT-Cre/Ai32 mice (n=11 cells).

H) Effect of photostimulation on steady-state V_m and AP properties during I_{inj}. Left, example pair of traces in a CA3PC under control conditions (black) and with photostimulation (blue). Dashed box indicates the time window for measurement of steady-state properties. Middle, steady-state membrane potential during the I_{inj} under control conditions and with photostimulation (blue); Wilcoxon test, p=0.109. Right, relative change in kinetic properties of steady-state APs by photostimulation of cholinergic axons (n=10 cells exhibiting regular steady-state AP firing). **p<0.01, one-sample Wilcoxon test compared to median=1.

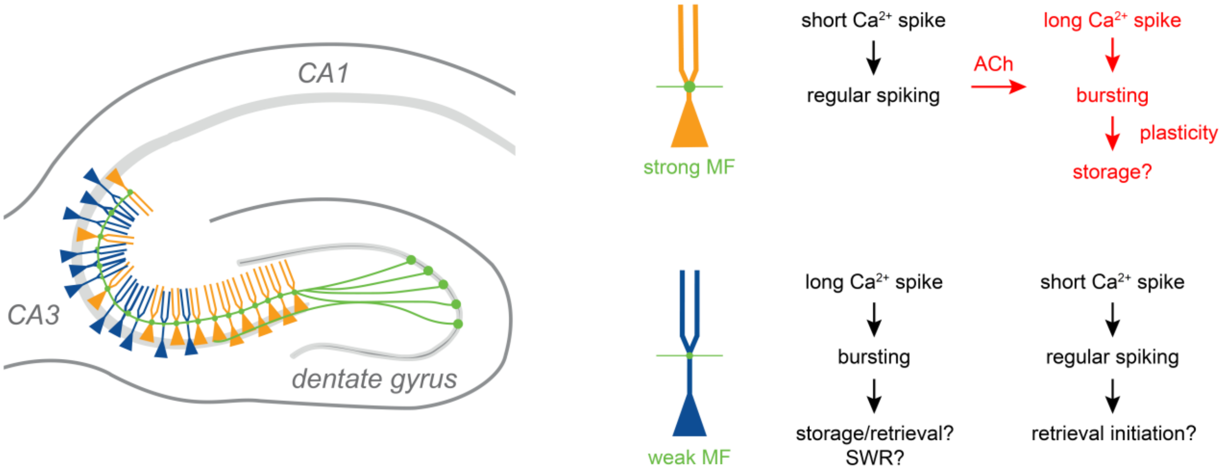

Figure S8. Cartoon illustrating the functional role and cholinergic regulation of cell subtype specific Ca^{2+} spike types.

Blue CA3PCs are mostly located in deeper layers of CA3a-b, on average have relatively longer primary apical trunks and fewer thorny excrescences (TEs) receiving input from granule cell MFs (green), and express long Ca^{2+} spikes and CSBs to strong widespread dendritic depolarization even under baseline conditions. Orange CA3PCs are distributed densely in proximal CA3c and extend to the superficial layer of distal CA3a-b, have short or multiple primary trunks and more abundant TEs, and express short Ca^{2+} spikes and no or only brief CSBs under baseline conditions. We hypothesize that these short Ca^{2+} spikes amplify synaptic depolarization and facilitate AP firing per se, but are not sufficient to drive robust synaptic plasticity of recurrent and/or entorhinal cortical synapses. An increase in cholinergic tone (e.g. during attentive behavioral states associated with learning) prolongs short Ca^{2+} spikes by modulation of K_A and K_M to allow long Ca^{2+} plateaus and CSBs, triggered by MF and/or EC input. We hypothesize that such prolonged plateaus (in concert with other cholinergic mechanisms facilitating plasticity) are required to induce rapid changes in synaptic weights by BTSP to encode novel information, possibly transmitted by dense MF inputs to these neurons.

The role of long Ca^{2+} spikes and CSBs could be different in deep distal long-shafted CA3PCs, where CSB firing even during low-ACh states (e.g. quiet immobility and slow-wave sleep) may reliably trigger activity in downstream neurons, for example during sharp-wave ripples (SWRs)(24). The role of the short Ca^{2+} spike component in these neurons is unclear but it might be evoked in higher order apical dendrites specifically by distal EC inputs to initiate AP firing without inducing long CSBs.

Supplemental References

1. Á. Magó, N. Kis, B. Lükő, J. K. Makara, Distinct dendritic Ca2+ spike forms produce opposing input-output transformations in rat CA3 pyramidal cells. *Elife* **10**, 74493 (2021).

2. S. Raus Balind, *et al.*, Diverse synaptic and dendritic mechanisms of complex spike burst generation in hippocampal CA3 pyramidal cells. *Nat. Commun.* **10**, 1859 (2019).

3. Q. Sun, *et al.*, Proximodistal Heterogeneity of Hippocampal CA3 Pyramidal Neuron Intrinsic Properties, Connectivity, and Reactivation during Memory Recall. *Neuron* **95**, 656-672.e3 (2017).

4. M. Kaneda, Y. Oyama, Y. Ikemoto, N. Akaike, Blockade of the voltage-dependent sodium current in isolated rat hippocampal neurons by tetrodotoxin and lidocaine. *Brain Res.* **484**, 348-351 (1989).

5. M. Madeja, Do neurons have a reserve of sodium channels for the generation of action potentials? A study on acutely isolated CA1 neurons from the guinea-pig hippocampus. *Eur. J. Neurosci.* **12**, 1-7 (2000).

6. F. M. Dreyfus, *et al.*, Selective T-type calcium channel block in thalamic neurons reveals channel redundancy and physiological impact of I(T)window. *J. of Neurosci.* **30,** 99-109 (2010).

7. W. Choe, *et al.*, TTA-P2 is a potent and selective blocker of T-type calcium channels in rat sensory neurons and a novel antinociceptive agent. *Mol. Pharmacol.* **80**, 900-910 (2011).

8. S. K. Mishra, K. Hermsmeyer, Selective inhibition of T-type Ca2+ channels by Ro 40-5967. *Circ. Res.* **75**, 144-148 (1994).

9. G. Mehrke, X. G. Zong, V. Flockerzi, F. Hofmann F, The Ca(++)-channel blocker Ro 40-5967 blocks differently T-type and L-type Ca++ channels. *J. Pharmacol. Exp. Ther.* **271**, 1483-1488 (1994).

10. L. Huang L, *et al.*, NNC 55-0396 [(1S,2S)-2-(2-(N-[(3-benzimidazol-2-yl)propyl]-N-methylamino)ethyl)-6-fluoro-1,2,3,4-tetrahydro-1-isopropyl-2-naphtyl cyclopropanecarboxylate dihydrochloride]: a new selective inhibitor of T-type calcium channels. *J. Pharmacol. Exp. Ther.* **309**, 193-199 (2004).

11. R. Newcomb R, *et al.*, Selective peptide antagonist of the class E calcium channel from the venom of the tarantula Hysterocrates gigas. *Biochemistry* **37**, 15353-15362 (1998).

12. D. R. Hillyard, *et al.*, A new Conus peptide ligand for mammalian presynaptic Ca2+ channels. *Neuron* **9**, 69-77 (1992).

13. T. Furukawa, *et al.*, Selectivities of dihydropyridine derivatives in blocking Ca(2+) channel subtypes expressed in Xenopus oocytes. *J. Pharmacol. Exp. Ther.* **291**, 464-473 (1999).

14. T. Furukawa, *et al.*, Differential blocking action of dihydropyridine Ca2+ antagonists on a T-type Ca2+ channel (alpha1G) expressed in Xenopus oocytes. *J. Cardiovasc. Pharmacol.* **45**, 241-246 (2005).

15. T. Kimm, B. P. Bean, Inhibition of A-type potassium current by the peptide toxin SNX-482. *J. of Neurosci.* **34**, 9182-9189 (2014).

16. H. Kametani, H. Kawamura, Alterations in acetylcholine release in the rat hippocampus during sleep-wakefulness detected by intracerebral dialysis. *Life Sci.* **47**, 421-426 (1990).

17. C. K. McIntyre, L. K. Marriott, P- E. Gold, Patterns of brain acetylcholine release predict individual differences in preferred learning strategies in rats. *Neurobiol. Learn. Mem.* **79**, 177-183 (2003).

18. K. Nail-Boucherie, N. Dourmap, R. Jaffard, J. Costentin, Contextual fear conditioning is associated with an increase of acetylcholine release in the hippocampus of rat. *Brain Res. Cogn. Brain Res.* **9**, 193-197 (2000).

19. G. Pepeu, M. G. Giovannini, Changes in acetylcholine extracellular levels during cognitive processes. *Learn. Mem.* **11**, 21-27 (2004).

20. Y. S. Mineur, M. R. Picciotto, How can I measure brain acetylcholine levels in vivo? Advantages and caveats of commonly used approaches. *J. Neurochem.* **167**, 3-15 (2023).

21. H. I. Yamamura, S. H. Snyder, Muscarinic cholinergic binding in rat brain. *Proc. Natl. Acad. Sci. U.S.A.* **71**, 1725-1729 (1974).

22. K. J. Kellar, A. M. Martino, D. P. Jr Hall, R. D. Schwartz, R. L. Taylor, High-affinity binding of [3H]acetylcholine to muscarinic cholinergic receptors. *J. of Neurosci.* **5**, 1577-1582 (1985).

23. N. L. Golding, H. Y. Jung, T. Mickus, N. Spruston, Dendritic calcium spike initiation and repolarization are controlled by distinct potassium channel subtypes in CA1 pyramidal neurons. *J. Neurosci.* 19, 8789-8798 (1999).

24. D. L. Hunt, D. Linaro, B. Si, S. Romani, N. Spruston, A novel pyramidal cell type promotes sharp-wave synchronization in the hippocampus. *Nat. Neurosci.* **21**, 985–995 (2018).
